# DNA Double Strand Break Repair and Its Control by Nucleosome Remodeling

**DOI:** 10.3389/fgene.2021.821543

**Published:** 2022-01-12

**Authors:** Leonhard Andreas Karl, Martina Peritore, Lorenzo Galanti, Boris Pfander

**Affiliations:** Resarch Group DNA Replication and Genome Integrity, Max Planck Institute of Biochemistry, Martinsried, Germany

**Keywords:** nucleosome remodeling, double strand break, DNA repair, DNA end resection, cell cycle, genome stability

## Abstract

DNA double strand breaks (DSBs) are repaired in eukaryotes by one of several cellular mechanisms. The decision-making process controlling DSB repair takes place at the step of DNA end resection, the nucleolytic processing of DNA ends, which generates single-stranded DNA overhangs. Dependent on the length of the overhang, a corresponding DSB repair mechanism is engaged. Interestingly, nucleosomes—the fundamental unit of chromatin—influence the activity of resection nucleases and nucleosome remodelers have emerged as key regulators of DSB repair. Nucleosome remodelers share a common enzymatic mechanism, but for global genome organization specific remodelers have been shown to exert distinct activities. Specifically, different remodelers have been found to slide and evict, position or edit nucleosomes. It is an open question whether the same remodelers exert the same function also in the context of DSBs. Here, we will review recent advances in our understanding of nucleosome remodelers at DSBs: to what extent nucleosome sliding, eviction, positioning and editing can be observed at DSBs and how these activities affect the DSB repair decision.

## Introduction

DNA double strand breaks are a highly toxic form of DNA damage, arising from intrinsic and extrinsic sources (Ciccia and Elledge, 2010). Eukaryotes are equipped with several mechanisms to repair DSBs, including non-homologous end joining (NHEJ), alternative end joining (alt-EJ), homologous recombination (HR) and single strand annealing (SSA) (Chang et al., 2017; Ranjha et al., 2018). Notably, these pathways do not only have different prerequisites (for example HR requiring a homologous donor sequence), but they also differ in the repair outcome and the potential to introduce genetic changes (such as mutations and chromosomal rearrangements). The cellular repair pathway decision is therefore critical for the survival of the affected cell or organism as well as for the stability of its genome (Symington and Gautier, 2011). Moreover, the fact that DSB repair is controlled by endogenous factors is a major limitation for genome editing strategies, which can nowadays involve efficient delivery of DSBs at the gene of interest, but often lead to a heterogenous outcome of the genome editing reaction across cell populations.

The cellular DSB repair pathway decision is made at the step of DNA end resection, the nucleolytic processing of DSB ends (Symington and Gautier, 2011; Cejka, 2015; Daley et al., 2015; Symington, 2016; Bonetti et al., 2018). Resection involves endo- and exonucleolytic cleavage of DNA ends that reveals 3′ single-stranded DNA overhangs. Notably, resection destroys the substrate for repair by NHEJ and increasing amounts of 3′ single-stranded DNA (ssDNA) predisposes for repair by different mechanisms (alt EJ < HR < SSA, Blier et al., 1993; Falzon et al., 1993; Ira et al., 2004). The enzymatic process of resection has been subject of excellent reviews in this issue and elsewhere (Symington and Gautier, 2011; Cejka and Symington, 2021; Elbakry and Löbrich, 2021; Sanchez et al., 2021). Here we focus on how resection and thereby the repair pathway decision is regulated by nucleosomes and nucleosome remodelers, enzymes that can evict, position and edit nucleosomes. For general reviews on how DNA damage triggers post-translational histone modifications, we refer to the following articles (Smeenk and van Attikum, 2013; Van and Santos, 2018).

**FIGURE 1:**
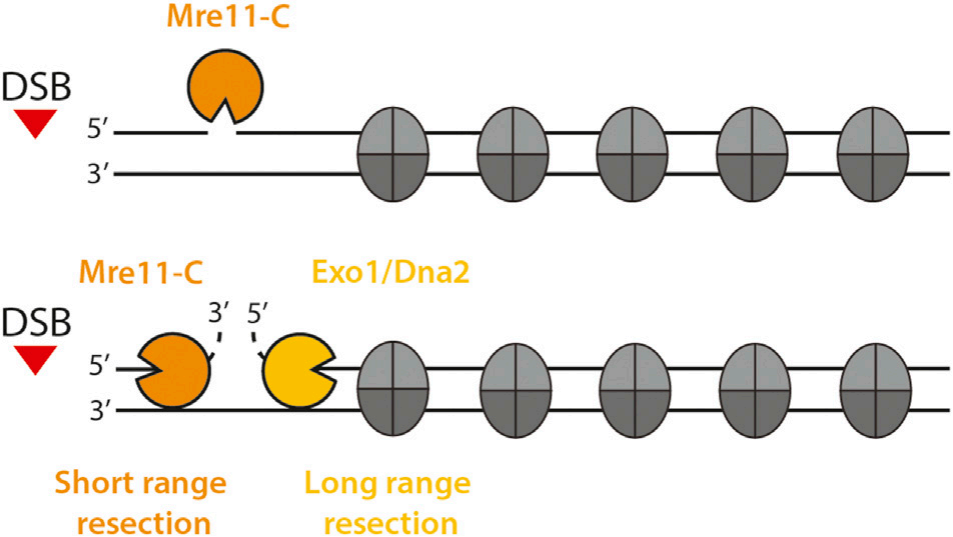
Eukaryotic DNA end resection in the chromatin context. DNA end resection is a two step process that can be divided into short-range resection (orange) and long-range resection (yellow). Mre11-C initiates short-range resection by nicking the 5′ terminated strand in proximity to the DSB via its endonuclease activity. Then, Mre11-C generates a short 3′ ssDNA overhang close to the DSB using its 3′-5′ exonuclease function. Exo1 and STR-Dna2 carry out long-range resection and extend the length of the resected ssDNA tract through chromatin.

Nucleosomes form obstacles to the resection nucleases (Figure 1). Initial short-range resection is carried out by the Mre11-complex (Mre11-C in the following, consisting of Mre11-Rad50-Xrs2 with the Sae2 activator in budding yeast, and analogously of MRE11-RAD50-NBS1 with CtIP in human) (Symington and Gautier, 2011; Cejka and Symington, 2021; Elbakry and Löbrich, 2021; Sanchez et al., 2021). Endonucleolytic cleavage by Mre11-C occurs preferentially within nucleosome-free linker DNA, suggesting that nucleosomal DNA is protected and/or that chromatin binding of Mre11-C is guided by nucleosomes (Mimitou et al., 2017; Wang et al., 2017). Moreover, the nucleases that carry out long-range resection are directly inhibited by the presence of nucleosomes: biochemical studies with yeast proteins have shown that the Exo1 exonuclease is unable to act on a nucleosome substrate and the combined helicase-endonuclease STR-Dna2 (Sgs1-Top3-Rmi1-Dna2) can only process nucleosomal DNA, if sufficient nucleosome-free DNA is present (Adkins et al., 2013). Therefore, nucleosomes are a barrier to the resection process and resection control factors are expected to modify the permeability of this barrier.

Nucleosome remodelers have received attention as regulators of DNA end resection and DSB repair pathway choice. These nucleosome remodelers are enzymes that in ATP-dependent fashion catalyze the breakage of histone-DNA-contacts within the nucleosome and translocate the DNA relative to histone proteins (Clapier and Cairns, 2009; Clapier et al., 2017). All eukaryotes possess several nucleosome remodelers - often in the form of multi-protein complexes - which are grouped into several sub-families according to the conservation of their ATPase subunit (Flaus et al., 2006). Biochemical and structural data suggest that the overall enzymatic mechanism of DNA translocation and breakage of DNA-histone contacts is highly related (Clapier and Cairns, 2009; Clapier et al., 2017) (with the potential exception of Fun30/SMARCAD1, see below). Nonetheless, studies on gene transcription and general chromatin organization have revealed that specific remodelers appear to have specific enzymatic activities, by which they slide, evict, position or edit nucleosomes (Clapier and Cairns, 2009; Clapier et al., 2017). Here, we will investigate whether such distinct roles can also be found in the context of DNA double strand breaks and how these activities may affect DSB repair. A natural focus of this review will be the budding yeast system, where remodelers have been studied comprehensively also in the context of DSBs, but we will additionally address whether the picture emerging from these studies is conserved in higher eukaryotes.

## Resection is Affected by Nucleosomes

In many eukaryotes, DNA end resection is carried out by three resection enzymes, Mre11-C, STR-Dna2 and Exo1, which act specifically at one of the two stages of the resection process (short-range resection/resection initiation and long-range resection/resection elongation, Figure 1). Notably, all three act by distinct molecular mechanisms and it is therefore unsurprising that nucleosomes have distinct effects on each of them. Mre11-C recognizes the DSB end either directly or through a DSB end-binding protein (most likely the end-binding factor Ku) and, after activation by Sae2/CtIP, induces a single-strand break on the 5′-strand (Sartori et al., 2007; Cannavo and Cejka, 2014; Anand et al., 2016; Deshpande et al., 2016; Reginato et al., 2017; Wang et al., 2017). From this point, bidirectional resection occurs: Mre11-C catalyzes 3′-5′ exonucleolytic resection towards the break, while Dna2 and Exo1 exonucleases catalyze long-range resection with 5′-3′ polarity into undamaged chromatin (Mimitou and Symington, 2008; Zhu et al., 2008; Cejka et al., 2010; Niu et al., 2010; Garcia et al., 2011; Shibata et al., 2014).

Preferential cleavage of linker DNA indicates that nucleosomal DNA may be refractory to endonucleolytic clipping by Mre11-C (Mimitou et al., 2017; Wang et al., 2017). However, nucleosomes per se are not a barrier to Mre11-C. Rather, it can slide or reach over nucleosomes (Myler et al., 2017; Wang et al., 2017). In cases where such bypass occurs, the nucleosome located between DSB and incision site could then potentially constitute a barrier to the 3′-5′ exonuclease activity of Mre11-C. Given the dual endo- and exonucleolytic activities of Mre11-C, this question has so far been difficult to address.

Long-range resection enzymes are even more strongly affected by the presence of nucleosomes. For example, *in vitro* studies have shown that Exo1 cannot resect through nucleosomes (Adkins et al., 2013), suggesting that additional activities are needed to overcome the chromatin barrier. Interestingly, changing nucleosome composition may be sufficient to allow Exo1-mediated resection. Incorporation of the H2A-variant H2A.Z decreases nucleosome stability and increases accessibility of nucleosomal DNA (Abbott et al., 2001; Zhang et al., 2005; Jin and Felsenfeld, 2007; Adkins et al., 2013; Watanabe et al., 2013; Lewis et al., 2021), which may allow Exo1 to bypass the nucleosomal barrier (Adkins et al., 2013).

In contrast to Exo1, the other long-range resection enzyme STR-Dna2 is in principle able to bypass nucleosomes. This may be due to a different enzymatic mechanism. While during long-range resection STR-Dna2 has the net effect of an exonuclease, STR-Dna2 utilizes the combined action of the Sgs1 helicase that unwinds DNA, followed by endonucleolytic cleavage of the emerging flap structure by Dna2 (Cejka et al., 2010; Niu et al., 2010). Apparently, the Sgs1 helicase motor is powerful enough to disrupt nucleosomes, allowing STR-Dna2 to resect nucleosomal DNA (Adkins et al., 2013). However, in order to carry out resection of nucleosomal DNA, STR-Dna2 will need as much as 300 bp of free DNA to be able to traverse through nucleosomes (Adkins et al., 2013). This distance is greater than the nucleosomal linker DNA-length and, consistently, STR-Dna2 is effectively inhibited by a nucleosomal array (Adkins et al., 2013).

Therefore, both long-range resection enzymes are blocked by chromatin and will require the activity of additional factors. One factor that could help to overcome the nucleosomal barrier is Mre11-C. Speculatively, Mre11-C could catalyze further endonucleolytic incisions downstream of the nucleosome from which long-range nucleases could (re-)initiate and thereby allow to bypass the nucleosome barrier. Currently, such an auxiliary role of Mre11-C in long-range resection lacks experimental support, but recent data suggest that short-range and long-range resection nucleases work in a coordinated fashion (Ceppi et al., 2020).

Alternatively, resection enzymes will need assistance by chromatin remodelers to get past nucleosomes and it is therefore important to consider how these enzymes may be able to modify the nucleosome barrier.

## Remodelers Have Distinct Roles in Chromatin Organization

Eukaryotes express several nucleosome remodelers (Flaus et al., 2006) and chromatin immunoprecipitation (ChIP) and related techniques have localized several of them to DSBs (Bantele et al., 2017; Bennett and Peterson, 2015; Bennett et al., 2013; Bird et al., 2002; Chai et al., 2005; Chen et al., 2012; Costelloe et al., 2012; Downs et al., 2004; Eapen et al., 2012; Gnugnoli et al., 2021; Lademann et al., 2017; Morrison et al., 2004; Shim et al., 2005; 2007; Tsukuda et al., 2005; van Attikum et al., 2004; 2007). This raises the question, whether these remodelers have distinct functions at DSBs or whether they act redundantly.

Nucleosome remodelers are found to be either single protein enzymes or multi-protein complexes. Historically, four major sub-families of remodelers have been proposed (Clapier and Cairns, 2009), but phylogenetic analysis based on sequence conservation of the catalytic ATPase subunits showed the existence of additional sub-families (Flaus et al., 2006). Five sub-families are found throughout eukaryotes – ISWI, SWI/SNF, CHD1, INO80 and Fun30/ETL. In contrast, ALC1, CHD7 and Mi2/NURD sub-families are not found throughout eukaryotes, with ALC1 and CHD7 orthologues specifically found in metazoans (Tong et al., 1998; Xue et al., 1998; Zhang et al., 1998; Ma et al., 2008; Bouazoune and Kingston, 2012). Table 1 summarizes the different remodeler sub-families with their putative catalytic activities and involvement in DSB repair.

**TABLE 1:** Overview of nucleosome remodeler sub-families and their members.

Family	Sub-family	Putative activity	*S. cerevisiae*	*H. sapiens* orthologues	Function at DSBs
Snf2-like	SWI/SNF	Nucleosome sliding/eviction	SWI/SNF	BAF	Delamarre et al. (2020), Hays et al. (2020), Hu et al. (2020), Kakarougkas et al. (2014), Kent et al. (2007), Lee et al. (2010), Meisenberg et al. (2019), Ogiwara et al. (2011), Peng et al. (2009), Peritore et al. (2021), Qi et al. (2015), Shim et al. (2005), Shim et al. (2007), Ui et al. (2014), Watanabe et al. (2014), Wiest et al. (2017)
			RSC	PBAF	
	ISWI	Nucleosome positioning	Isw1a	ACF	Casari et al. (2021), Delamarre et al. (2020), Helfricht et al. (2013), Lan et al. (2010), Nakamura et al. (2011), Pessina and Lowndes, (2014), Sánchez-Molina et al. (2011), Sheu et al. (2010), Smeenk et al. (2012), Toiber et al. (2013), Vidi et al. (2014), Xiao et al. (2009)
				CHRAC	
			Isw1b	NoRC	
				RSF	
			Isw2	WICH	
				NURF	
				CERF	
	CHD-I	Nucleosome positioning	Chd1	CHD1, CHD2	Delamarre et al. (2020), Gnugnoli et al. (2021), Kari et al. (2016), Luijsterburg et al. (2016), Zhou et al. (2018)
	CHD-II	?	-	Mi-2/NuRD	Chou et al. (2010), Goodarzi et al. (2011), Larsen et al. (2010), Luijsterburg et al. (2012), Pan et al. (2012), Polo et al. (2010), Qi et al. (2016), Smeenk et al. (2010), Smith et al. (2018), Spruijt et al. (2016)
	CHD-III	?	-	CHD6, CHD7, CHD8, CHD9	Rother et al. (2020)
	ALC1	?	-	ALC1	Ahel et al. (2009), Blessing et al. (2020), Juhász et al. (2020), Sellou et al. (2016)
Swr1-like	INO80	Nucleosome editing	INO80	INO80	Adkins et al. (2013), Alatwi and Downs, (2015), Bennett et al. (2013), Brahma et al. (2017), Chen et al. (2012), Downs et al. (2004), Kalocsay et al. (2009), Lademann et al. (2017), Morillo-Huesca et al. (2010), Morrison et al. (2004), Oberbeckmann et al. (2021b), Papamichos-Chronakis et al. (2006), Tsukuda et al. (2005), van Attikum et al. (2004), van Attikum et al. (2007)
		Nucleosome positioning	SWR1	SRCAP	
				TRAPP/Tip60	
	Fun30/ETL	?	Fun30	SMARCAD1	Bantele et al. (2017), Chen et al. (2012), Costelloe et al. (2012), Densham et al. (2016), Eapen et al. (2012)

Nucleosome remodelers are grouped into two families based on conservation of the ATPase subunit: Snf2-like and Swr1-like. Both families have several sub-families.

Snf2-like: The SWI/SNF (switch/sucrose non-fermentable) sub-family consists of two members in budding yeast - SWI/SNF and RSC (remodels the structure of chromatin) – as well as in human – BAF and PBAF. For human BAF variant complexes can be found harbouring ATPase subunit paralogs (Mittal and Roberts, 2020). The ISWI (imitation switch) sub-family in yeast contains 3 active complexes – Isw1a, Isw1b, Isw2 - that combine 2 different catalytic subunits - Isw1 and Isw2 - with different sets of proteins. For humans the setup with 2 catalytic subunits is similar, but with a higher number of different complexes: ACF, CHRAC, NoRC, RSF, WICH, NURF, CERF (Aydin et al., 2014). The CHD (chromodomain helicase DNA-binding) sub-family has a single member in yeast – Chd1 - and 3 subfamilies with in total 9 members in human: CHD1-2, CHD3-5 – forming NuRD/Mi-2 complex and CHD6-9 (Marfella and Imbalzano, 2007). The ALC1 sub-family carries a macrodomain for poly(ADP-ribose)-binding instead of a chromodomain and is found in human (Ahel et al., 2009).

Swr1-like: The INO80 (inositol requiring) sub-family has two members in yeast: INO80 and SWR1. In humans again there is additional complexity of this sub-family with INO80, SRCAP and TRAPP/Tip60 complexes (Willhoft and Wigley, 2020). The Fun30/ETL sub-family contains Fun30 in yeast and SMARCAD1 in human (Bantele and Pfander, 2019). Even though nucleosome remodelers appear to follow a highly similar enzymatic mechanism, they appear to exhibit distinct activities in chromatin organization. These putative activities are given along studies showing possible functions at DNA double strand breaks.

**FIGURE 2:**
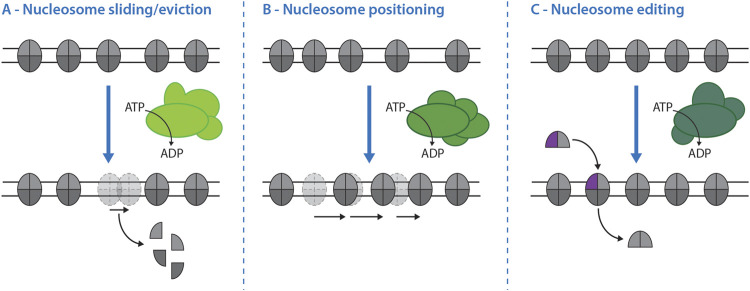
Nucleosome remodeler activities and their effects on chromatin. The activity of different nucleosome remodelers (shades of green) can result in three principal effects on nucleosomes. **(A)**–Nucleosome sliding and eviction. While all remodelers have the propensity to slide nucleosomes, eviction of nucleosomes from double-stranded DNA is catalyzed mainly by the SWI/SNF sub-family of nucleosome remodelers. **(B)**–Nucleosome positioning. Some nucleosome remodelers have the ability to slide and position nuclesomes on DNA in a controlled fashion that leads to the formation of regularly spaced arrays. In the budding yeast system this activity is catalyzed mainly by ISW1a-, ISW1b-, Chd1 and INO80-complexes. **(C)**–Nucleosome editing. Nucleosome editing is defined as the exchange of canonical histones (grey) for non-canonical histone variants, like H2A.Z (purple), within the nucleosome and vice versa. In budding yeast H2A/H2A.Z exchange is performed by the INO80 sub-family of remodelers: the SWR1-complex catalyzes the incorporation of H2A.Z-H2B dimers, while the INO80-C is thought to catalyze the reverse reaction.

**FIGURE 3:**
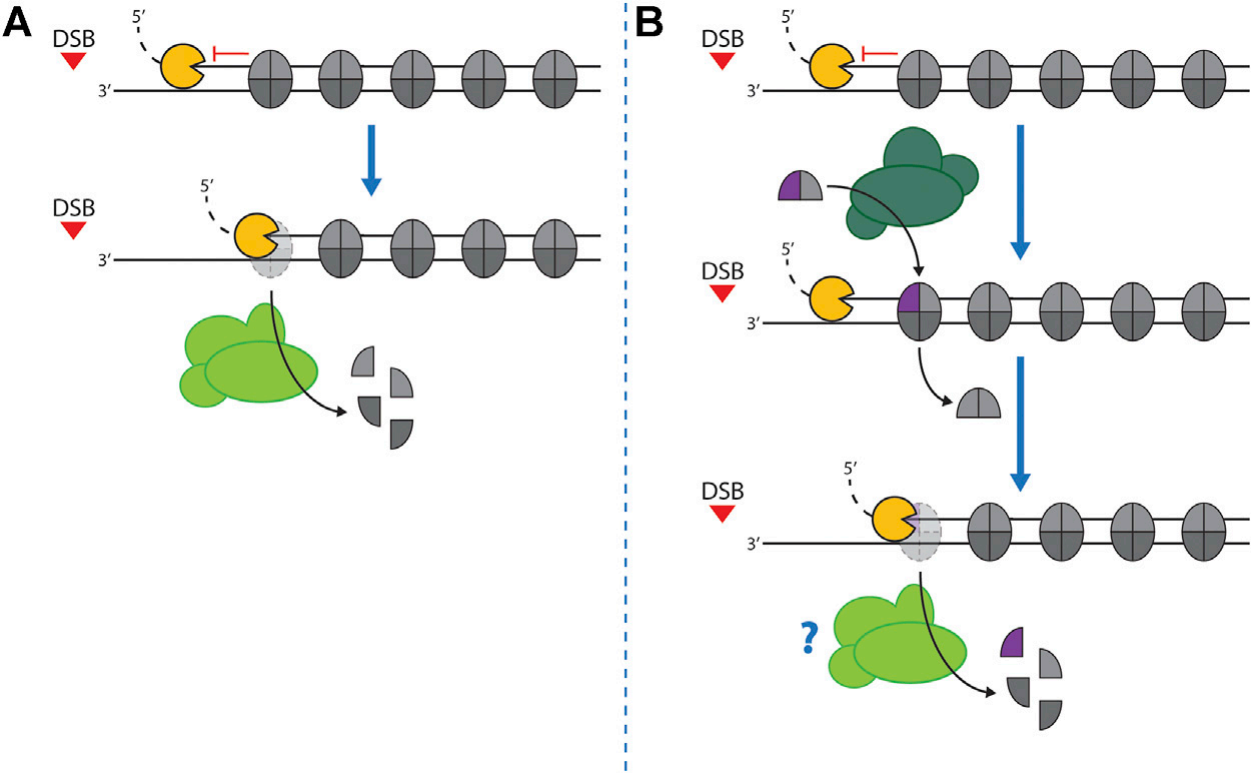
Nucleosome eviction at DSBs. **(A)**–Resection nucleases (Exo1/Dna2) are inhibited by the presence of nucleosomes. Thus, eviction of nucleosomes from dsDNA is required to facilitate resection. This reaction may be catalyzed by nucleosome remodelers with evicting activity (light green). Moreover, binding of the Mre11-C to the DSB ends might be inhibited by nucleosomes (not shown). Therefore, eviction by nucleosome remodelers might be additionally required also for resection initiation. **(B)**–Incorporation of H2A.Z (purple) into nucleosomes by nucleosome remodelers with editing activity (dark green) leads to a reduced stability of nucleosomes. H2A.Z-containing nucleosomes may therefore be directly evicted by long-range resection nucleases, but nucleosome remodelers with evicting activity (light green) may be additionally involved (see “?”).

It seems expedient to group remodelers not only by evolutionary conservation, but also by functional similarity (Figure 2, Table 1). *In vitro* and *in vivo* we can discriminate at least three activities of nucleosome remodelers: 1) sliding/eviction leads to movement of nucleosomes along DNA that can even result in the removal of the entire nucleosome (Figure 2A); 2) positioning involves movement of nucleosomes to form regularly spaced nucleosomal arrays (Figure 2B); 3) editing involves the exchange of histones (commonly H2A-H2B dimers) to alter the composition of nucleosomes (Clapier and Cairns, 2009; Clapier et al., 2017). Based on studies of genome-wide chromatin organization, we currently think that SWI/SNF sub-family complexes (SWI/SNF and RSC in yeast) act as major sliding/eviction enzymes, that ISWI and CHD1 sub-family remodelers as well as INO80-C act as positioning enzymes and that INO80 sub-family complexes (SWR1 and INO80 in yeast) catalyze editing (Table 1, Clapier and Cairns, 2009; Clapier et al., 2017). In the following, we will investigate whether nucleosome remodelers carry out the same activities at DSBs.

## Nucleosome Eviction and Resection are Coupled

With nucleosomes forming a barrier to resection, nucleosome eviction is the most straight-forward solution to allow spreading of resection into chromatin (Figure 3). Indeed, nucleosomes are lost around DSBs in the region where resection occurs (Bantele and Pfander, 2019; Chen et al., 2008; Mimitou et al., 2017; Tsukuda et al., 2005; 2009; van Attikum et al., 2007). While it was proposed that nucleosomes may associate in some form with resected, single-stranded DNA to form single-stranded nucleosomes (Adkins et al., 2017; Huang et al., 2018), a dedicated study did not find evidence to support wide-spread association of nucleosomes with single-stranded DNA *in vivo* (Peritore et al., 2021). But how do nucleosomes become evicted and how do sliding/evicting nucleosome remodelers of the SWI/SNF sub-family facilitate this eviction (Figure 3A)? In budding yeast, the SWI/SNF and RSC complexes are specifically recruited to DSBs (Chai et al., 2005; Shim et al., 2005, 2007; Kent et al., 2007; Liang et al., 2007; Bennett et al., 2013; Bennett and Peterson, 2015; Wiest et al., 2017), suggesting that they may act during DSB repair or signaling. To interrogate the function of SWI/SNF and RSC, deletion of non-essential subunits or conditional depletion of the essential catalytic subunits have been used. Interestingly, interfering with either SWI/SNF or RSC function induced a defect already in the association of Mre11-C with DSBs (Shim et al., 2007; Wiest et al., 2017), suggesting that these remodelers could act at an early stage of DSB repair. Notably, under single-mutant conditions, resection and DSB repair were found to be delayed or reduced, but not abolished. Recently, experimental conditions were established that allowed to simultaneously induce the degradation of the ATPase subunits of both SWI/SNF and RSC (Peritore et al., 2021). Under these double mutant conditions, we find that nucleosome eviction and resection are both blocked (Peritore et al., 2021). This indicates that 1) SWI/SNF and RSC are redundantly required for DNA end resection and that 2) resection and nucleosome eviction are intrinsically coupled. Altogether, these data are consistent with SWI/SNF and RSC complexes playing a major role as nucleosome evictors also in the context of DSBs. The single mutant data (Shim et al., 2007; Wiest et al., 2017), which showed defects in recruitment of Mre11-C, suggest that both complexes play an early role and evict or move DSB-proximal nucleosomes to allow binding of Mre11-C as well as resection initiation. Whether SWI/SNF or RSC influence Mre11-C activity, endonucleolytic clipping in particular, remains to be tested.

These findings raise the question of how these nucleosome remodelers sense the presence of a DSB and become recruited to DSB-proximal chromatin at such an early stage. Notably, RSC and SWI/SNF localize to the proximity of DSBs independently of each other and follow different recruitment kinetics, suggesting that they recognize DSBs by different mechanisms. RSC is recruited to a DSB within 10 min and thereby precedes resection initiation (Chai et al., 2005). While its recruitment kinetics are therefore similar to those of Mre11-C (Shim et al., 2007), we currently do not understand which signal is being recognized by RSC. SWI/SNF in contrast shows significantly slower recruitment (Chai et al., 2005) that depends on nucleosome modifications. Specifically, histone acetylation is recognized by SWI/SNF and appears to lead to its DSB recruitment, consistent with the presence of several acetylation-binding bromodomains in the SWI/SNF complex (Bennett and Peterson, 2015; Cheng et al., 2021). Notably, the histone acetyltransferase NuA4 is specifically recruited to DSBs and this recruitment was shown to depend on Mre11-C (Cheng et al., 2021). The fact that SWI/SNF was found to be required for recruitment of Mre11-C, but at the same time also dependent on Mre11-C activity (Shim et al., 2005, 2007; Wiest et al., 2017) is not necessarily a contradiction, but could suggest the presence of a positive feedback loop that promotes resection initiation.

Biochemical data suggest that long-range resection should be particularly dependent on nucleosome eviction (Adkins et al., 2013). Consistently, SWI/SNF appears to stimulate long range resection (Wiest et al., 2017), but this has yet to be correlated with nucleosome eviction. Altogether, these data show that RSC and SWI/SNF complexes promote DNA end resection in budding yeast and likely do so by acting as nucleosome evictors. Detailed biochemical and cell biological analysis will however be needed to pinpoint exactly at which steps of DNA end resection these nucleosome remodelers act. These studies also need to account for the fact that RSC and SWI/SNF may also influence the long-range chromatin response to DSBs (on the 10 kb–1 Mb range). Indeed, γH2A - the long-range chromatin mark of DSBs - was found to be reduced in RSC mutants (Kent et al., 2007; Shim et al., 2007), but it is unclear whether this effect relates to nucleosome eviction.

Lastly, SWI/SNF is also required later during HR, as SWI/SNF mutants show defects in synapsis and strand invasion (Chai et al., 2005). It is currently unclear whether this is due to defects in resection, due to a second “late” role in HR or due to long-range chromatin changes on the broken chromosome.

Nucleosome eviction appears to be conserved in human remodeler complexes. Human BAF and PBAF complexes are recruited to sites of DSBs (Park et al., 2006; Hays et al., 2020). Moreover, they appear to promote resection, possibly by acting on the Mre11-C activator CtIP (Hays et al., 2020). This suggests an early role in resection and it will be interesting to investigate whether this function is linked to nucleosome eviction.

While SWI/SNF and RSC are the major players in nucleosome eviction, it could be possible that also other nucleosome remodelers evict nucleosomes during DSB repair and resection. In particular, the INO80 complex has been linked to the eviction of nucleosomes at sites of transcription and DSBs as well (Tsukuda et al., 2005; van Attikum et al., 2007; Qiu et al., 2020), but given several functions of INO80 during DSB repair (see below) this activity is particularly challenging to ascertain. Additionally, whatever this INO80 complex function is, it appears to act differently from SWI/SNF and RSC complexes (Peritore et al., 2021). In all, we therefore conclude that 1) histone eviction occurs at DSBs, that 2) it is critical for DSB resection and repair and that 3) it appears to be mediated by the major cellular eviction activities of the SWI/SNF sub-family complexes.

## The Role of Nucleosome Positioning at Double Strand Breaks Remains to be Determined

Nucleosomes are positioned in a non-random fashion throughout the genome. In particular, a specific organization is seen at sites of transcribed genes, where a nucleosome-free region marks or neighbors the transcription start site, followed by regularly spaced nucleosomal arrays (Yuan et al., 2005; Weiner et al., 2010; Baldi et al., 2020). Positioning remodelers are responsible for the characteristic spacing of nucleosomes within such nucleosome arrays (Baldi et al., 2020). To generate the specific spacing of nucleosomes within the array, positioning remodelers use intrinsic ruler mechanisms as well as sensing of DNA shapes (Yamada et al., 2011; Krietenstein et al., 2016; Oberbeckmann et al., 2021a; Oberbeckmann et al., 2021b). The generation of nucleosome arrays has been extensively studied in budding yeast, where a combination of *in vitro* and *in vivo* studies suggests that four remodelers – Chd1, ISW1a, ISW2 and INO80 – can specifically position nucleosomes to form nucleosome arrays (Gkikopoulos et al., 2011; Krietenstein et al., 2016; Ocampo et al., 2016; Kubik et al., 2019; Oberbeckmann et al., 2021b). Importantly, these remodelers also sense the presence of barrier-factors bound at specific sites in the genome to which the array is aligned to or “phased” (Eaton et al., 2010; Li et al., 2015; Krietenstein et al., 2016; Kubik et al., 2018; Rossi et al., 2018). Typical barrier factors are DNA-binding factors, like the abundant general regulatory factors Abf1, Rap1 or Reb1 in budding yeast or genome organizing factors like CTCF in mammals or Phaser in flies (Fu et al., 2008; Wiechens et al., 2016; Baldi et al., 2018). Importantly, recent *in vitro* work suggests that also DSBs are sensed as a barrier-factor by nucleosome remodelers and guide the formation of nucleosome arrays (Oberbeckmann et al., 2021a).

The finding that regularly spaced nucleosome arrays can form around DSBs in *in vitro* systems raises two questions: do remodelers position nucleosomes to form arrays around DSBs also *in vivo* and would such arrays promote DNA end resection? Experimentally, nucleosome positioning is typically investigated using micrococcal nuclease (MNase), which cleaves preferentially non-nucleosomal DNA. Several studies that used MNase to investigate nucleosome localization around a single DSB showed eviction of DSB-proximal nucleosomes, but came to different conclusions as to whether DSB-distal nucleosomes would shift their position (Kent et al., 2007; Shim et al., 2007; Tsabar et al., 2016). While these results are seemingly contradictory, this may simply be due to the fact that results from a single DSB are difficult to interpret. For example the newly formed array can be indistinguishable from the initial nucleosome positions, if the DSB and initial barrier factor are located at the same position. To overcome these limitations, a recent study utilized the *PHO5* gene, with its well characterized nucleosomal array and found evidence for eviction of the break-proximal nucleosome as well as repositioning of further distal nucleosomes (Tripuraneni et al., 2021). Further studies will need to show whether repositioned nucleosomes are indeed aligned to the DSB and whether the DSB itself or DSB-associated proteins serve as barrier. Furthermore, studies need to identify, if arrays are generated by positioning remodelers Chd1, ISW1a, ISW2 or INO80.

Interestingly, several studies in both yeast and human cells, point towards a function of these specific remodelers in promoting homologous recombination (Lan et al., 2010; Nakamura et al., 2011; Smeenk et al., 2012; Toiber et al., 2013; Kari et al., 2016; Zhou et al., 2018; Rother et al., 2020; Casari et al., 2021; Gnugnoli et al., 2021). In particular, remodelers of ISWI, CHD1 and CHD7 sub-families appear to be recruited to sites of DNA damage and to stimulate resection (Smeenk et al., 2012; Toiber et al., 2013; Kari et al., 2016; Delamarre et al., 2020; Rother et al., 2020; Gnugnoli et al., 2021). The precise mechanism by which these remodelers promote resection and HR is however uncertain. Moreover, even if these remodelers established nucleosome arrays around DSBs, it is at this point entirely unclear whether such arrays will have a positive function in DSB repair or whether they are simply a consequence of the enzymatic mechanism of positioning remodelers (Baldi et al., 2018). Therefore, despite first hints that nucleosome arrays could form in the proximity of DSBs, the role of positioning remodelers in DSB repair still needs to be determined.

## Nucleosome Editing and H2A.Z Exchange Guide Double Strand Break Repair

Nucleosome editing describes the activity of exchanging canonical histone subunits with non-canonical histone variants and vice versa (Das and Tyler, 2013; Venkatesh and Workman, 2015). Nucleosome remodelers can facilitate editing by catalyzing the exchange of histone dimers. In eukaryotes, several histone variants exist primarily for H2A and H3 (Draizen et al., 2016; Talbert and Henikoff, 2010; 2017; 2021). While the principal mechanism for the incorporation of H3 and its variants is via *de novo* assembly of nucleosomes, H2A and its variants can be incorporated into existing nucleosomes by H2A-H2B dimer exchange (reviewed in Luger et al., 2012). In this chapter, we will therefore concentrate on nucleosome editing of H2A. Nucleosome editing has been extensively studied in budding yeast, where two H2A variants exist: H2A (which includes features of H2A.X) and H2A.Z (Santisteban et al., 2000). The SWR1 complex catalyzes the incorporation of H2A.Z-H2B dimers (Krogan et al., 2003; Mizuguchi et al., 2004). Furthermore, the INO80 complex is thought to catalyze the reverse reaction, the exchange of H2A.Z-H2B with H2A-H2B dimers (Papamichos-Chronakis et al., 2011; Brahma et al., 2017). This model of INO80 function is based on the principal finding that deletion of the H2A.Z gene *HTZ1* genetically suppresses many phenotypes of mutants deficient in INO80 function (Lademann et al., 2017; Papamichos-Chronakis et al., 2006; 2011).

The SWR1 complex is the prototypical nucleosome editing remodeler: mechanistically, it is able to translocate short stretches of DNA with no changes in nucleosome position, which then allows H2A-H2B dimers to be exchanged for H2A.Z-H2B dimers (Wu et al., 2009; Luk et al., 2010; Ranjan et al., 2015; Willhoft et al., 2018; Singh et al., 2019). In budding yeast, the SWR1 complex incorporates H2A.Z into chromatin around DSBs, as indicated by 1) the recruitment of SWR1 to DSB sites (van Attikum et al., 2007; Morillo-Huesca et al., 2010) and 2) a transient increase in H2A.Z occupancy in the DSB-surrounding chromatin shortly after DSB induction (Kalocsay et al., 2009). A transiently increased incorporation of H2A.Z into DSB-proximal chromatin was observed also in human cells (Xu et al., 2012; Nishibuchi et al., 2014; Alatwi and Downs, 2015; Gursoy-Yuzugullu et al., 2015). Compared to canonical nucleosomes, H2A.Z-containing nucleosomes are more labile (Abbott et al., 2001; Zhang et al., 2005; Jin and Felsenfeld, 2007) suggesting that their presence will promote DNA end resection. Consistently, yeast cells lacking H2A.Z show a pronounced resection defect (Kalocsay et al., 2009; Lademann et al., 2017). In contrast, the absence of SWR1 causes a much milder resection phenotype (van Attikum et al., 2007; Chen et al., 2012; Adkins et al., 2013). These data suggest that either 1) H2A.Z becomes incorporated at DSB sites by an SWR1-independent mechanism or that 2) H2A.Z-incorporation into DSB-surrounding chromatin is not a major regulator of resection and that H2A.Z regulates resection by means independent from its incorporation in DSB-surrounding chromatin.

If H2A.Z-incorporation into DSB-proximal chromatin promotes resection, there are two putative mechanisms by which it could do so. First, the aforementioned reduction of nucleosome stability may allow remodelers or even resection nucleases to bypass and evict H2A.Z-containing nucleosomes (Adkins et al., 2013). Second, H2A.Z could serve as binding platform for associated factors (Xu et al., 2012) as has been shown for nucleotide excision repair (Yu et al., 2013). Binding of factors to H2A.Z or SUMO-modified H2A.Z is for example thought to lead to relocalization of DSBs to the nuclear periphery (Nagai et al., 2008; Kalocsay et al., 2009; Oza et al., 2009; Horigome et al., 2014). Relocalization of DSBs is also observed in Drosophila, where heterochromatic DSBs are first brought to the periphery of the heterochromatic domain (Chiolo et al., 2011) and then to the nuclear pore complex (Ryu et al., 2015). Similarly, in mammalian cells DSB relocation to discrete clusters in the periphery of heterochromatin has been observed (Jakob et al., 2011; Tsouroula et al., 2016; Schrank et al., 2018), but a connection between DSB relocation and H2A.Z has not been shown so far. Therefore, nucleosome editing and H2A.Z incorporation are used to regulate DSB repair, but the underlying molecular mechanisms warrant further investigation.

The importance of nucleosome editing for DSB repair raises the question whether H2A.Z incorporation becomes reversed at some point. Indeed, studies in budding yeast have shown that the INO80 complex is not only recruited to DSBs (Downs et al., 2004; Morrison et al., 2004; van Attikum et al., 2004; Bennett et al., 2013), but that it also counteracts H2A.Z incorporation (Papamichos-Chronakis et al., 2011). Also in human cells H2A.Z is removed from chromatin surrounding DSB sites (Xu et al., 2012; Nishibuchi et al., 2014; Alatwi and Downs, 2015; Gursoy-Yuzugullu et al., 2015; Clouaire et al., 2018). While INO80’s role as nucleosome editing and H2A.Z removal enzyme was initially controversial (Papamichos-Chronakis et al., 2011; Watanabe et al., 2013; Jeronimo et al., 2015; Tramantano et al., 2016; Wang et al., 2016; Watanabe and Peterson, 2016), recent structural work showed that besides its nucleosome positioning activity, the INO80 complex may be able to catalyze translocation of short stretches of DNA without nucleosome sliding, consistent with histone dimer exchange activity (Ayala et al., 2018; Eustermann et al., 2018). This suggests that at DSBs INO80 may have at least two activities: 1) a nucleosome positioning activity (see above) and 2) a nucleosome editing activity (Papamichos-Chronakis et al., 2006; Alatwi and Downs, 2015; Brahma et al., 2017; Lademann et al., 2017). Consistent with INO80 antagonizing the SWR1 complex and removing H2A.Z from chromatin, mutants deficient in INO80 complex function accumulate H2A.Z around DSBs (Papamichos-Chronakis et al., 2006; Alatwi and Downs, 2015; Lademann et al., 2017). The dual remodeling activity of the INO80 complex complicates the interpretation of *ino80* mutant phenotypes. To overcome this issue, deletion of the H2A.Z gene *HTZ1* has been used*,* because it suppresses phenotypes arising from an H2A.Z removal defect. Using this approach, an H2A.Z removal function of the INO80 complex was found to promote the formation of the Rad51 nucleo-protein filament downstream of resection (Lademann et al., 2017). In contrast, a resection-promoting function of the INO80 complex was found to be independent of H2A.Z (Lademann et al., 2017) and therefore unrelated to nucleosome editing. Moreover, also in human cells, nucleosome editing by the INO80 complex is important for DSB repair and acts after DNA end resection (Alatwi and Downs, 2015). Taken together, a picture emerges whereby nucleosome editing and H2A.Z incorporation by the SWR1 complex is involved in regulation of DNA end resection in yeast, while generally and throughout eukaryotes H2A.Z removal in DSB-surrounding chromatin is important for DSB repair, but likely acts only after resection.

## Fun30/SMARCAD1 Promote Resection by Antagonizing Resection-Inhibitory Factors

Fun30 (from budding yeast), ETL1 (from mouse) and SMARCAD1 (from human) are the prototypical members of a sub-family of nucleosome remodelers that is evolutionary conserved throughout eukaryotes (Clark et al., 1992; Adra et al., 2000; Flaus et al., 2006). Historically they have not been considered major nucleosome remodelers and their molecular mechanisms have not yet been entirely elucidated (Bantele and Pfander, 2019). Recently, a study by the Luger lab suggested that SMARCAD1 evicts and also assembles entire nucleosomes by a mechanism that involves unique contacts between remodeler and nucleosome (Markert et al., 2021). Work with yeast Fun30 suggests that it can slide nucleosomes and mediate histone dimer exchange (Awad et al., 2010).

A key function of yeast Fun30 and human SMARCAD1 appears to be the stimulation of long-range resection (Chen et al., 2012; Costelloe et al., 2012; Eapen et al., 2012). For example, in budding yeast cells lacking Fun30, long-range resection of a non-repairable DSB is 2-3-fold slower than in WT cells (Eapen et al., 2012; Bantele et al., 2017). Accordingly, *fun30* mutants scored similarly to mutants deficient in the long-range resection nucleases, when they were initially found in screens for resection-dependent repair of DSBs (Chen et al., 2012; Costelloe et al., 2012). Moreover, an evolutionary conserved pathway facilitates recruitment of Fun30 to sites of DNA end resection. This pathway requires the 9-1-1 complex as recruitment platform at the ssDNA-dsDNA junction and is activated during cell cycle phases (S-M phase), when also resection is activated (Chen et al., 2016; Bantele et al., 2017).

In contrast, Fun30 did not stimulate Exo1’s ability to resect through a nucleosome in an *in vitro* system (Adkins et al., 2013). This finding raises the possibility that a crucial factor was missing from these reconstituted systems. Consistently, *fun30* mutant phenotypes can be suppressed by the additional depletion of the resection inhibitor Rad9 from yeast cells (Chen et al., 2012; Bantele et al., 2017). These data indicate a functional antagonism between Fun30 and Rad9. Notably, also in human cells SMARCAD1 acts as resection activator, while the Rad9 orthologue 53BP1 is a resection inhibitor (Lazzaro et al., 2008; Bunting et al., 2010; Bothmer et al., 2011; Costelloe et al., 2012; Densham et al., 2016), suggesting that the antagonism of both factors is conserved throughout eukaryotic evolution (please see (Sanchez et al., 2021)) in this issue for a detailed review on the interaction between 53BP1 and BRCA1 in the DSB repair decision). Notably, Rad9, 53BP1, as well as the fission yeast orthologue Crb2 associate with chromatin and have all been shown to bind to nucleosomes, where they recognize specific histone modifications (Huyen et al., 2004; Nakamura et al., 2004; Sanders et al., 2004; Wysocki et al., 2005; Botuyan et al., 2006; Du et al., 2006; Toh et al., 2006; Grenon et al., 2007; Hammet et al., 2007; Fradet-Turcotte et al., 2013; Wilson et al., 2016; Hu et al., 2017; Kilic et al., 2019). Rad9 orthologues appear to recognize distinct histone marks, but in each case two or more histone marks are bound (reviewed in Marini et al., 2019; Panier and Boulton, 2014), suggesting that Rad9 orthologues are multivalent histone binders. We therefore hypothesize that both Fun30 and Rad9 influence DSB-surrounding chromatin in an antagonistic fashion and that Fun30 specifically acts on Rad9-bound nucleosomes (Bantele and Pfander, 2019).

**FIGURE 4:**
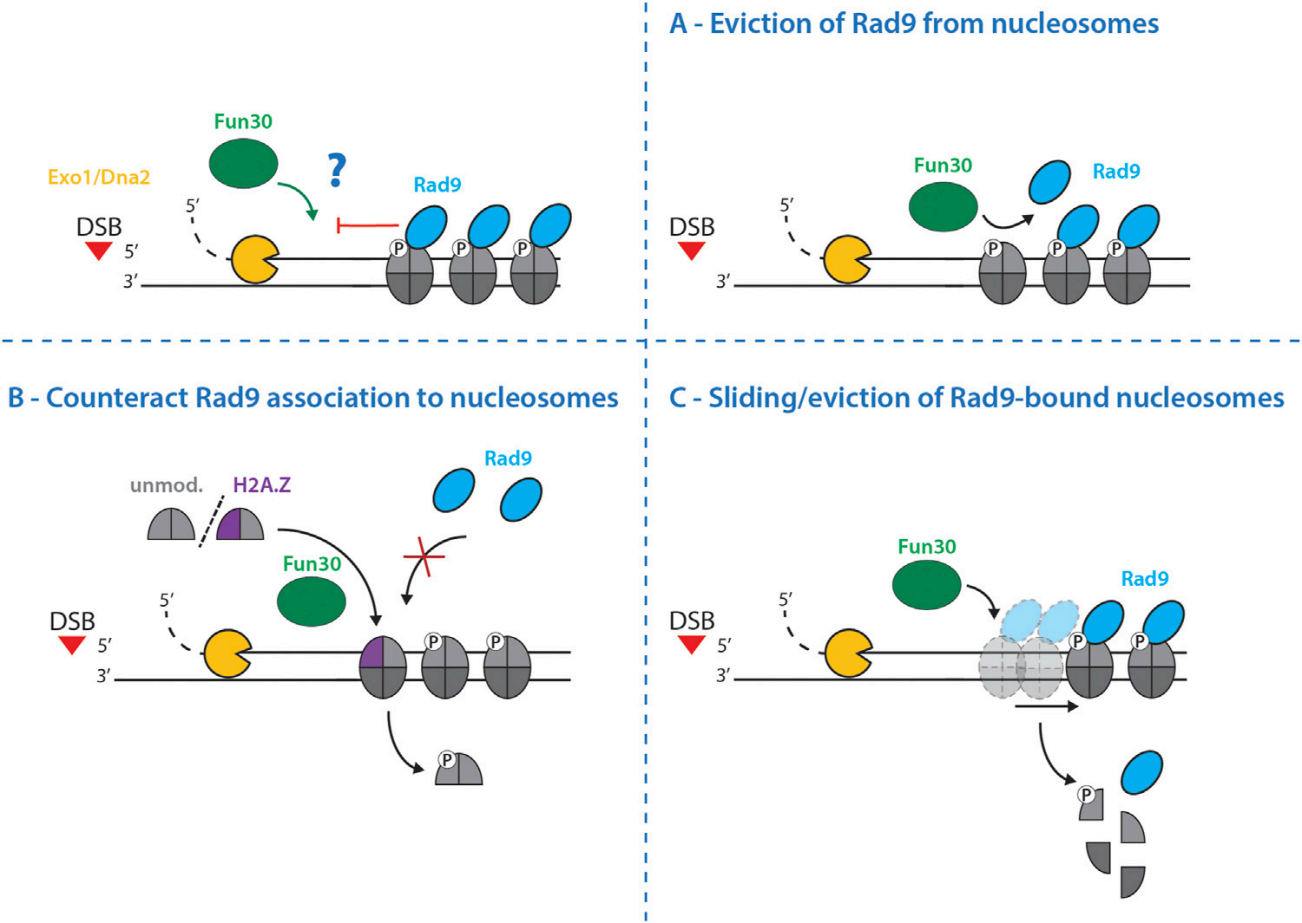
Potential mechanisms by which Fun30 may promote resection. Long-range resection is controlled by the antagonism between the resection-promoting nucleosome remodeler Fun30 and the resection-inhibiting nucleosome binder Rad9. The precise mechanism of this antagonistic relationship is still elusive, but the following models are possible: **(A)**–Fun30 directly removes Rad9 from nucleosomes thereby removing the factor inhibiting resection. **(B)**–Fun30 counteracts Rad9 association with nucleosomes by exchanging histone dimers. It either incorporates histones lacking modifications necessary for Rad9 association – for example unmodified H2A, missing phosphorylation on S129 (γH2A), or the histone variant H2A.Z; both of which eliminate Rad9 binding sites. **(C)**–Fun30 slides and/or evicts Rad9-bound nucleosomes, freeing the DNA from the resection-inhibitory effects of Rad9 to allow the subsequent resection.

In budding yeast cells lacking both Fun30 and Rad9, resection and nucleosome eviction are fully functional (Peritore et al., 2021), suggesting that Fun30 is not required to overcome the general nucleosome barrier and that it is not the essential nucleosome evictor at DSBs. Alternatively, Fun30 may rather catalyze the direct removal of Rad9 from nucleosomes (Figure 4A) or it may counteract Rad9 association with nucleosomes by catalyzing histone dimer exchange which may remove one or more binding site(s) for Rad9 (Figure 4B). Lastly, it is possible that Fun30 slides or even entirely evicts Rad9-bound nucleosomes (Figure 4C). Given that Rad9 and Fun30 antagonize each other on multiple levels, including also the competition for binding to the scaffold protein Dpb11 (Granata et al., 2010; Pfander and Diffley, 2011; Bantele et al., 2017), future biochemical and structural studies will be needed to reveal the mechanism by which Fun30 promotes DNA end resection.

Also human SMARCAD1 antagonizes 53BP1. Depletion of SMARCAD1 stabilizes 53BP1 around DSB sites (Densham et al., 2016). However, resection regulation in human cells is more complex compared to yeast as besides SMARCAD1 a second resection promoting factor exist, the BRCA1-BARD1 complex (reviewed in Densham and Morris, 2019; Sanchez et al., 2021). BRCA1-BARD1 form an E3 ubiquitin-ligase complex that mediates ubiquitylation of H2A (Kalb et al., 2014; Densham et al., 2016; Leung et al., 2017; Nakamura et al., 2019). BRCA1-BARD1 is likely to act upstream of SMARCAD1, as ubiquitin-modified H2A promotes SMARCAD1 binding to nucleosomes around DSBs (Densham et al., 2016). Therefore, SMARCAD1 function has to be seen in the context of post-translational histone modifications, which affect DSB-surrounding chromatin. DSB-localized SMARCAD1 may also become post-translationally modified itself, including phosphorylation by the ATM kinase and ubiquitylation by the RING1 ubiquitin ligase (Chakraborty et al., 2018), which appears to activate the pro-resection function of SMARCAD1. These factors need to be taken into consideration for biochemical studies that ultimately will allow to understand whether the Fun30/SMARCAD1 sub-family remodelers facilitate resection by nucleosomes sliding and eviction, positioning or editing and whether it acts on nucleosomes or rather on nucleosome-associated proteins.

## Conclusion

In all, we think that previous studies collectively indicate that nucleosome remodelers may serve similar roles during DSB repair as during gene transcription with nucleosome eviction, editing and potentially even positioning taking place at DSBs. Knowledge of the specific activities of individual nucleosome remodelers and of their redundancies thereby offers the potential to get to grips with chromatin changes occurring at DSBs. Moreover, we think that studies of DSB resection and repair may be generally inspired by analogies to gene transcription. Both processes appear to be similarly affected by the presence of chromatin, with nucleosomes forming a dynamic barrier and nucleosome remodelers facilitating its bypass.

Importantly, while nucleosomes clearly form a barrier to the resection nucleases, nucleosome remodelers equip cells with multiple ways to overcome this barrier. In this review, we have outlined several putative mechanisms of how bypass may occur. These include eviction, sliding and editing of nucleosomes. While we are still only beginning to understand how the nucleosome barrier is overcome, a key future question will be which bypass mechanism is chosen in which cellular scenario. Importantly, the nucleosome barrier and its dynamic nature offers additional possibilities to regulate resection and DSB repair. Moreover, critical factors of the DSB repair decision, such as 53BP1 and BRCA1, are proteins that bind and modify nucleosomes. Therefore, we propose that convergence of resection-regulatory pathways on nucleosomes is a central part of the cellular DSB repair decision.

## References

[B1] Abbott D. W. Ivanova V. S. Wang X. Bonner W. M. Ausió J. (2001). Characterization of the Stability and Folding of H2A.Z Chromatin Particles. J. Biol. Chem. 276, 41945–41949. 10.1074/jbc.M108217200 11551971

[B2] Adkins N. L. Niu H. Sung P. Peterson C. L. (2013). Nucleosome Dynamics Regulates DNA Processing. Nat. Struct. Mol. Biol. 20, 836–842. 10.1038/nsmb.2585 23728291 PMC3711194

[B3] Adkins N. L. Swygert S. G. Kaur P. Niu H. Grigoryev S. A. Sung P. (2017). Nucleosome-like, Single-Stranded DNA (ssDNA)-Histone Octamer Complexes and the Implication for DNA Double Strand Break Repair*. J. Biol. Chem. 292, 5271–5281. 10.1074/jbc.m117.776369 28202543 PMC5392674

[B4] Adra C. N. Donato J.-L. Badovinac R. Syed F. Kheraj R. Cai H. (2000). SMARCAD1, a Novel Human Helicase Family-Defining Member Associated with Genetic Instability: Cloning, Expression, and Mapping to 4q22-Q23, a Band Rich in Breakpoints and Deletion Mutants Involved in Several Human Diseases. Genomics 69, 162–173. 10.1006/geno.2000.6281 11031099

[B5] Ahel D. Hořejší Z. Wiechens N. Polo S. E. Garcia-Wilson E. Ahel I. (2009). Poly(ADP-ribose)-dependent Regulation of DNA Repair by the Chromatin Remodeling Enzyme ALC1. Science 325, 1240–1243. 10.1126/science.1177321 19661379 PMC3443743

[B6] Alatwi H. E. Downs J. A. (2015). Removal of H2A.Z by INO 80 Promotes Homologous Recombination. EMBO Rep. 16, 986–994. 10.15252/embr.201540330 26142279 PMC4552491

[B7] Anand R. Ranjha L. Cannavo E. Cejka P. (2016). Phosphorylated CtIP Functions as a Co-factor of the MRE11-RAD50-NBS1 Endonuclease in DNA End Resection. Mol. Cel 64, 940–950. 10.1016/j.molcel.2016.10.017 27889449

[B8] Awad S. Ryan D. Prochasson P. Owen-Hughes T. Hassan A. H. (2010). The Snf2 Homolog Fun30 Acts as a Homodimeric ATP-dependent Chromatin-Remodeling Enzyme. J. Biol. Chem. 285, 9477–9484. 10.1074/jbc.M109.082149 20075079 PMC2843198

[B9] Ayala R. Willhoft O. Aramayo R. J. Wilkinson M. McCormack E. A. Ocloo L. (2018). Structure and Regulation of the Human INO80-Nucleosome Complex. Nature 556, 391–395. 10.1038/s41586-018-0021-6 29643506 PMC5937682

[B10] Aydin Ö. Z. Vermeulen W. Lans H. (2014). ISWI Chromatin Remodeling Complexes in the DNA Damage Response. Cell Cycle 13, 3016–3025. 10.4161/15384101.2014.956551 25486562 PMC4615051

[B11] Baldi S. Jain D. S. Harpprecht L. Zabel A. Scheibe M. Butter F. (2018). Genome-wide Rules of Nucleosome Phasing in Drosophila. Mol. Cel 72, 661–672. e4. 10.1016/j.molcel.2018.09.032 30392927

[B12] Baldi S. Korber P. Becker P. B. (2020). Beads on a String-Nucleosome Array Arrangements and Folding of the Chromatin Fiber. Nat. Struct. Mol. Biol. 27, 109–118. 10.1038/s41594-019-0368-x 32042149

[B13] Bantele S. C. Ferreira P. Gritenaite D. Boos D. Pfander B. (2017). Targeting of the Fun30 Nucleosome Remodeller by the Dpb11 Scaffold Facilitates Cell Cycle-Regulated DNA End Resection. eLife Sci. 6, e21687. 10.7554/eLife.21687 PMC5300703 28063255

[B14] Bantele S. C. S. Pfander B. (2019). Nucleosome Remodeling by Fun30SMARCAD1 in the DNA Damage Response. Front. Mol. Biosci. 6. 10.3389/fmolb.2019.00078 PMC6737033 31555662

[B15] Bennett G. Papamichos-Chronakis M. Peterson C. L. (2013). DNA Repair Choice Defines a Common Pathway for Recruitment of Chromatin Regulators. Nat. Commun. 4, 2084. 10.1038/ncomms3084 23811932 PMC3731036

[B16] Bennett G. Peterson C. L. (2015). SWI/SNF Recruitment to a DNA Double-Strand Break by the NuA4 and Gcn5 Histone Acetyltransferases. DNA Repair 30, 38–45. 10.1016/j.dnarep.2015.03.006 25869823 PMC4425604

[B17] Bird A. W. Yu D. Y. Pray-Grant M. G. Qiu Q. Harmon K. E. Megee P. C. (2002). Acetylation of Histone H4 by Esa1 Is Required for DNA Double-Strand Break Repair. Nature 419, 411–415. 10.1038/nature01035 12353039

[B18] Blessing C. Mandemaker I. K. Gonzalez-Leal C. Preisser J. Schomburg A. Ladurner A. G. (2020). The Oncogenic Helicase ALC1 Regulates PARP Inhibitor Potency by Trapping PARP2 at DNA Breaks. Mol. Cel 80, 862–875.e6. 10.1016/j.molcel.2020.10.009 33275888

[B19] Blier P. R. Griffith A. J. Craft J. Hardin J. A. (1993). Binding of Ku Protein to DNA. Measurement of Affinity for Ends and Demonstration of Binding to Nicks. J. Biol. Chem. 268, 7594–7601. 10.1016/s0021-9258(18)53216-6 8463290

[B20] Bonetti D. Colombo C. V. Clerici M. Longhese M. P. (2018). Processing of DNA Ends in the Maintenance of Genome Stability. Front. Genet. 9, 390. 10.3389/fgene.2018.00390 30258457 PMC6143663

[B21] Bothmer A. Robbiani D. F. Di Virgilio M. Bunting S. F. Klein I. A. Feldhahn N. (2011). Regulation of DNA End Joining, Resection, and Immunoglobulin Class Switch Recombination by 53BP1. Mol. Cel 42, 319–329. 10.1016/j.molcel.2011.03.019 PMC3142663 21549309

[B22] Botuyan M. V. Lee J. Ward I. M. Kim J.-E. Thompson J. R. Chen J. (2006). Structural Basis for the Methylation State-specific Recognition of Histone H4-K20 by 53BP1 and Crb2 in DNA Repair. Cell 127, 1361–1373. 10.1016/j.cell.2006.10.043 17190600 PMC1804291

[B23] Bouazoune K. Kingston R. E. (2012). Chromatin Remodeling by the CHD7 Protein Is Impaired by Mutations that Cause Human Developmental Disorders. Proc. Natl. Acad. Sci. U S A. 109, 19238–19243. 10.1073/pnas.1213825109 23134727 PMC3511097

[B24] Brahma S. Udugama M. I. Kim J. Hada A. Bhardwaj S. K. Hailu S. G. (2017). INO80 Exchanges H2A.Z for H2A by Translocating on DNA Proximal to Histone Dimers. Nat. Commun. 8, 15616. 10.1038/ncomms15616 28604691 PMC5472786

[B25] Bunting S. F. Callén E. Wong N. Chen H.-T. Polato F. Gunn A. (2010). 53BP1 Inhibits Homologous Recombination in Brca1-Deficient Cells by Blocking Resection of DNA Breaks. Cell 141, 243–254. 10.1016/j.cell.2010.03.012 20362325 PMC2857570

[B26] Cannavo E. Cejka P. (2014). Sae2 Promotes dsDNA Endonuclease Activity within Mre11-Rad50-Xrs2 to Resect DNA Breaks. Nature 514, 122–125. 10.1038/nature13771 25231868

[B27] Casari E. Gobbini E. Gnugnoli M. Mangiagalli M. Clerici M. Longhese M. P. (2021). Dpb4 Promotes Resection of DNA Double-Strand Breaks and Checkpoint Activation by Acting in Two Different Protein Complexes. Nat. Commun. 12, 4750. 10.1038/s41467-021-25090-9 34362907 PMC8346560

[B28] Cejka P. Cannavo E. Polaczek P. Masuda-Sasa T. Pokharel S. Campbell J. L. (2010). DNA End Resection by Dna2-Sgs1-RPA and its Stimulation by Top3-Rmi1 and Mre11-Rad50-Xrs2. Nature 467, 112–116. 10.1038/nature09355 20811461 PMC3089589

[B29] Cejka P. (2015). DNA End Resection: Nucleases Team up with the Right Partners to Initiate Homologous Recombination. J. Biol. Chem. 290, 22931–22938. 10.1074/jbc.r115.675942 26231213 PMC4645618

[B30] Cejka P. Symington L. S. (2021). DNA End Resection: Mechanism and Control. Annu. Rev. Genet. 55, 285–307. 10.1146/annurev-genet-071719-020312 34813349

[B31] Ceppi I. Howard S. M. Kasaciunaite K. Pinto C. Anand R. Seidel R. (2020). CtIP Promotes the Motor Activity of DNA2 to Accelerate Long-Range DNA End Resection. Proc. Natl. Acad. Sci. USA 117, 8859–8869. 10.1073/pnas.2001165117 32241893 PMC7183222

[B32] Chai B. Huang J. Cairns B. R. Laurent B. C. (2005). Distinct Roles for the RSC and Swi/Snf ATP-dependent Chromatin Remodelers in DNA Double-Strand Break Repair. Genes Dev. 19, 1656–1661. 10.1101/gad.1273105 16024655 PMC1176001

[B33] Chakraborty S. Pandita R. K. Hambarde S. Mattoo A. R. Charaka V. Ahmed K. M. (2018). SMARCAD1 Phosphorylation and Ubiquitination Are Required for Resection during DNA Double-Strand Break Repair. iScience 2, 123–135. 10.1016/j.isci.2018.03.016 29888761 PMC5993204

[B34] Chang H. H. Y. Pannunzio N. R. Adachi N. Lieber M. R. (2017). Non-homologous DNA End Joining and Alternative Pathways to Double-Strand Break Repair. Nat. Rev. Mol. Cel Biol 18, 495–506. 10.1038/nrm.2017.48 PMC7062608 28512351

[B35] Chen C.-C. Carson J. J. Feser J. Tamburini B. Zabaronick S. Linger J. (2008). Acetylated Lysine 56 on Histone H3 Drives Chromatin Assembly after Repair and Signals for the Completion of Repair. Cell 134, 231–243. 10.1016/j.cell.2008.06.035 18662539 PMC2610811

[B36] Chen X. Cui D. Papusha A. Zhang X. Chu C.-D. Tang J. (2012). The Fun30 Nucleosome Remodeller Promotes Resection of DNA Double-Strand Break Ends. Nature 489, 576–580. 10.1038/nature11355 22960743 PMC3640768

[B37] Chen X. Niu H. Yu Y. Wang J. Zhu S. Zhou J. (2016). Enrichment of Cdk1-Cyclins at DNA Double-Strand Breaks Stimulates Fun30 Phosphorylation and DNA End Resection. Nucleic Acids Res. 44, 2742–2753. 10.1093/nar/gkv1544 26801641 PMC4824098

[B38] Cheng X. Côté V. Côté J. (2021). NuA4 and SAGA Acetyltransferase Complexes Cooperate for Repair of DNA Breaks by Homologous Recombination. Plos Genet. 17, e1009459. 10.1371/journal.pgen.1009459 34228704 PMC8284799

[B39] Chiolo I. Minoda A. Colmenares S. U. Polyzos A. Costes S. V. Karpen G. H. (2011). Double-Strand Breaks in Heterochromatin Move outside of a Dynamic HP1a Domain to Complete Recombinational Repair. Cell 144, 732–744. 10.1016/j.cell.2011.02.012 21353298 PMC3417143

[B40] Chou D. M. Adamson B. Dephoure N. E. Tan X. Nottke A. C. Hurov K. E. (2010). A Chromatin Localization Screen Reveals Poly (ADP Ribose)-Regulated Recruitment of the Repressive Polycomb and NuRD Complexes to Sites of DNA Damage. Proc. Natl. Acad. Sci. 107, 18475–18480. 10.1073/pnas.1012946107 20937877 PMC2972950

[B41] Ciccia A. Elledge S. J. (2010). The DNA Damage Response: Making it Safe to Play with Knives. Mol. Cel 40, 179–204. 10.1016/j.molcel.2010.09.019 PMC2988877 20965415

[B42] Clapier C. R. Cairns B. R. (2009). The Biology of Chromatin Remodeling Complexes. Annu. Rev. Biochem. 78, 273–304. 10.1146/annurev.biochem.77.062706.153223 19355820

[B43] Clapier C. R. Iwasa J. Cairns B. R. Peterson C. L. (2017). Mechanisms of Action and Regulation of ATP-dependent Chromatin-Remodelling Complexes. Nat. Rev. Mol. Cel Biol 18, 407–422. 10.1038/nrm.2017.26 PMC8127953 28512350

[B44] Clark M. W. Zhong W. W. Keng T. Storms R. K. Barton A. Kaback D. B. (1992). Identification of aSaccharomyces Cerevisiae Homolog of theSNF2 Transcrioptional Regulator in the DNA Sequence of an 8·6 Kb Region in theLTE1-CYS1 Interval on the Left Arm of Chormosome I. Yeast 8, 133–145. 10.1002/yea.320080208 1561836

[B45] Clouaire T. Rocher V. Lashgari A. Arnould C. Aguirrebengoa M. Biernacka A. (2018). Comprehensive Mapping of Histone Modifications at DNA Double-Strand Breaks Deciphers Repair Pathway Chromatin Signatures. Mol. Cel 72, 250–262.e6. 10.1016/j.molcel.2018.08.020 PMC6202423 30270107

[B46] Costelloe T. Louge R. Tomimatsu N. Mukherjee B. Martini E. Khadaroo B. (2012). The Yeast Fun30 and Human SMARCAD1 Chromatin Remodellers Promote DNA End Resection. Nature 489, 581–584. 10.1038/nature11353 22960744 PMC3493121

[B47] Daley J. M. Niu H. Miller A. S. Sung P. (2015). Biochemical Mechanism of DSB End Resection and its Regulation. DNA Repair 32, 66–74. 10.1016/j.dnarep.2015.04.015 25956866 PMC4522330

[B48] Das C. Tyler J. K. (2012). Histone Exchange and Histone Modifications during Transcription and Aging. Biochim. Biophys. Acta (Bba) - Gene Regul. Mech. 1819, 332–342. 10.1016/j.bbagrm.2011.08.001 PMC3981540 24459735

[B49] Delamarre A. Barthe A. de la Roche Saint-André C. Luciano P. Forey R. Padioleau I. (2020). MRX Increases Chromatin Accessibility at Stalled Replication Forks to Promote Nascent DNA Resection and Cohesin Loading. Mol. Cel 77, 395–410. e3. 10.1016/j.molcel.2019.10.029 31759824

[B50] Densham R. M. Garvin A. J. Stone H. R. Strachan J. Baldock R. A. Daza-Martin M. (2016). Human BRCA1-BARD1 Ubiquitin Ligase Activity Counteracts Chromatin Barriers to DNA Resection. Nat. Struct. Mol. Biol. 23, 647–655. 10.1038/nsmb.3236 27239795 PMC6522385

[B51] Densham R. M. Morris J. R. (2019). Moving Mountains-The BRCA1 Promotion of DNA Resection. Front. Mol. Biosci. 6. 10.3389/fmolb.2019.00079 PMC6733915 31552267

[B52] Deshpande R. A. Lee J.-H. Arora S. Paull T. T. (2016). Nbs1 Converts the Human Mre11/Rad50 Nuclease Complex into an Endo/Exonuclease Machine Specific for Protein-DNA Adducts. Mol. Cel 64, 593–606. 10.1016/j.molcel.2016.10.010 27814491

[B53] Downs J. A. Allard S. Jobin-Robitaille O. Javaheri A. Auger A. Bouchard N. (2004). Binding of Chromatin-Modifying Activities to Phosphorylated Histone H2A at DNA Damage Sites. Mol. Cel 16, 979–990. 10.1016/j.molcel.2004.12.003 15610740

[B54] Draizen E. J. Shaytan A. K. Mariño-Ramírez L. Talbert P. B. Landsman D. Panchenko A. R. (2016). HistoneDB 2.0: a Histone Database with Variants-An Integrated Resource to Explore Histones and Their Variants. Database 2016, baw014. 10.1093/database/baw014 26989147 PMC4795928

[B55] Du L.-L. Nakamura T. M. Russell P. (2006). Histone Modification-dependent and -independent Pathways for Recruitment of Checkpoint Protein Crb2 to Double-Strand Breaks. Genes Dev. 20, 1583–1596. 10.1101/gad.1422606 16778077 PMC1482479

[B56] Eapen V. V. Sugawara N. Tsabar M. Wu W.-H. Haber J. E. (2012). The *Saccharomyces cerevisiae* Chromatin Remodeler Fun30 Regulates DNA End Resection and Checkpoint Deactivation. Mol. Cel Biol 32, 4727–4740. 10.1128/MCB.00566-12 PMC3486187 23007155

[B57] Eaton M. L. Galani K. Kang S. Bell S. P. MacAlpine D. M. (2010). Conserved Nucleosome Positioning Defines Replication Origins. Genes Dev. 24, 748–753. 10.1101/gad.1913210 20351051 PMC2854390

[B58] Elbakry A. Löbrich M. (2021). Homologous Recombination Subpathways: A Tangle to Resolve. Front. Genet. 12, 723847. 10.3389/fgene.2021.723847 34408777 PMC8365153

[B59] Eustermann S. Schall K. Kostrewa D. Lakomek K. Strauss M. Moldt M. (2018). Structural Basis for ATP-dependent Chromatin Remodelling by the INO80 Complex. Nature 556, 386–390. 10.1038/s41586-018-0029-y 29643509 PMC6071913

[B60] Falzon M. Fewell J. W. Kuff E. L. (1993). EBP-80, a Transcription Factor Closely Resembling the Human Autoantigen Ku, Recognizes Single- to Double-Strand Transitions in DNA. J. Biol. Chem. 268, 10546–10552. 10.1016/s0021-9258(18)82233-5 8486707

[B61] Flaus A. Martin D. M. A. Barton G. J. Owen-Hughes T. (2006). Identification of Multiple Distinct Snf2 Subfamilies with Conserved Structural Motifs. Nucleic Acids Res. 34, 2887–2905. 10.1093/nar/gkl295 16738128 PMC1474054

[B62] Fradet-Turcotte A. Canny M. D. Escribano-Díaz C. Orthwein A. Leung C. C. Y. Huang H. (2013). 53BP1 Is a Reader of the DNA-Damage-Induced H2A Lys 15 Ubiquitin Mark. Nature 499, 50–54. 10.1038/nature12318 23760478 PMC3955401

[B63] Fu Y. Sinha M. Peterson C. L. Weng Z. (2008). The Insulator Binding Protein CTCF Positions 20 Nucleosomes Around its Binding Sites across the Human Genome. Plos Genet. 4, e1000138. 10.1371/journal.pgen.1000138 18654629 PMC2453330

[B64] Garcia V. Phelps S. E. L. Gray S. Neale M. J. (2011). Bidirectional Resection of DNA Double-Strand Breaks by Mre11 and Exo1. Nature 479, 241–244. 10.1038/nature10515 22002605 PMC3214165

[B65] Gkikopoulos T. Schofield P. Singh V. Pinskaya M. Mellor J. Smolle M. (2011). A Role for Snf2-Related Nucleosome-Spacing Enzymes in Genome-wide Nucleosome Organization. Science 333, 1758–1760. 10.1126/science.1206097 21940898 PMC3428865

[B66] Gnugnoli M. Casari E. Longhese M. P. (2021). The Chromatin Remodeler Chd1 Supports MRX and Exo1 Functions in Resection of DNA Double-Strand Breaks. Plos Genet. 17, e1009807. 10.1371/journal.pgen.1009807 34520455 PMC8462745

[B67] Goodarzi A. A. Kurka T. Jeggo P. A. (2011). KAP-1 Phosphorylation Regulates CHD3 Nucleosome Remodeling during the DNA Double-Strand Break Response. Nat. Struct. Mol. Biol. 18, 831–839. 10.1038/nsmb.2077 21642969

[B68] Granata M. Lazzaro F. Novarina D. Panigada D. Puddu F. Abreu C. M. (2010). Dynamics of Rad9 Chromatin Binding and Checkpoint Function Are Mediated by its Dimerization and Are Cell Cycle-Regulated by CDK1 Activity. Plos Genet. 6, e1001047. 10.1371/journal.pgen.1001047 20700441 PMC2916856

[B69] Grenon M. Costelloe T. Jimeno S. O'Shaughnessy A. FitzGerald J. Zgheib O. (2007). Docking onto Chromatin via theSaccharomyces Cerevisiae Rad9 Tudor Domain. Yeast 24, 105–119. 10.1002/yea.1441 17243194

[B70] Gursoy-Yuzugullu O. Ayrapetov M. K. Price B. D. (2015). Histone chaperone Anp32e removes H2A.Z from DNA double-strand breaks and promotes nucleosome reorganization and DNA repair. Proc. Natl. Acad. Sci. USA 112, 7507–7512. 10.1073/pnas.1504868112 26034280 PMC4475971

[B71] Hammet A. Magill C. Heierhorst J. Jackson S. P. (2007). Rad9 BRCT Domain Interaction with Phosphorylated H2AX Regulates the G1 Checkpoint in Budding Yeast. EMBO Rep. 8, 851–857. 10.1038/sj.embor.7401036 17721446 PMC1973948

[B72] Hays E. Nettleton E. Carter C. Morales M. Vo L. Passo M. (2020). The SWI/SNF ATPase BRG1 Stimulates DNA End Resection and Homologous Recombination by Reducing Nucleosome Density at DNA Double Strand Breaks and by Promoting the Recruitment of the CtIP Nuclease. Cell Cycle 19, 3096–3114. 10.1080/15384101.2020.1831256 33044911 PMC7714457

[B73] Helfricht A. Wiegant W. Thijssen P. Vertegaal A. Luijsterburg M. van Attikum H. (2013). Remodeling and Spacing Factor 1 (RSF1) Deposits Centromere Proteins at DNA Double-Strand Breaks to Promote Non-homologous End-Joining. Cell Cycle 12, 3070–3082. 10.4161/cc.26033 23974106 PMC3875681

[B74] Horigome C. Oma Y. Konishi T. Schmid R. Marcomini I. Hauer M. H. (2014). SWR1 and INO80 Chromatin Remodelers Contribute to DNA Double-Strand Break Perinuclear Anchorage Site Choice. Mol. Cel 55, 626–639. 10.1016/j.molcel.2014.06.027 25066231

[B75] Hu K. Li Y. Wu W. Xie L. Yan H. Cai Y. (2020). ATM‐Dependent Recruitment of BRD7 Is Required for Transcriptional Repression and DNA Repair at DNA Breaks Flanking Transcriptional Active Regions. Adv. Sci. 7, 2000157. 10.1002/advs.202000157 PMC7578904 33101843

[B76] Hu Q. Botuyan M. V. Cui G. Zhao D. Mer G. (2017). Mechanisms of Ubiquitin-Nucleosome Recognition and Regulation of 53BP1 Chromatin Recruitment by RNF168/169 and RAD18. Mol. Cel 66, 473–487.e9. 10.1016/j.molcel.2017.04.009 PMC5523955 28506460

[B77] Huang T.-H. Fowler F. Chen C.-C. Shen Z.-J. Sleckman B. Tyler J. K. (2018). The Histone Chaperones ASF1 and CAF-1 Promote MMS22L-TONSL-Mediated Rad51 Loading onto ssDNA during Homologous Recombination in Human Cells. Mol. Cel 69, 879–892.e5. 10.1016/j.molcel.2018.01.031 PMC5843376 29478807

[B78] Huyen Y. Zgheib O. DiTullio Jr R. A. Jr Gorgoulis V. G. Zacharatos P. Petty T. J. (2004). Methylated Lysine 79 of Histone H3 Targets 53BP1 to DNA Double-Strand Breaks. Nature 432, 406–411. 10.1038/nature03114 15525939

[B79] Ira G. Pellicioli A. Balijja A. Wang X. Fiorani S. Carotenuto W. (2004). DNA End Resection, Homologous Recombination and DNA Damage Checkpoint Activation Require CDK1. Nature 431, 1011–1017. 10.1038/nature02964 15496928 PMC4493751

[B80] Jakob B. Splinter J. Conrad S. Voss K.-O. Zink D. Durante M. (2011). DNA Double-Strand Breaks in Heterochromatin Elicit Fast Repair Protein Recruitment, Histone H2AX Phosphorylation and Relocation to Euchromatin. Nucleic Acids Res. 39, 6489–6499. 10.1093/nar/gkr230 21511815 PMC3159438

[B81] Jeronimo C. Watanabe S. Kaplan C. D. Peterson C. L. Robert F. (2015). The Histone Chaperones FACT and Spt6 Restrict H2A.Z from Intragenic Locations. Mol. Cel 58, 1113–1123. 10.1016/j.molcel.2015.03.030 PMC4475440 25959393

[B82] Jin C. Felsenfeld G. (2007). Nucleosome Stability Mediated by Histone Variants H3.3 and H2A.Z. Genes Dev. 21, 1519–1529. 10.1101/gad.1547707 17575053 PMC1891429

[B83] Juhász S. Smith R. Schauer T. Spekhardt D. Mamar H. Zentout S. (2020). The Chromatin Remodeler ALC1 Underlies Resistance to PARP Inhibitor Treatment. Sci. Adv. 6, eabb8626. 10.1126/sciadv.abb8626 33355125 PMC11206534

[B84] Kakarougkas A. Ismail A. Chambers A. L. Riballo E. Herbert A. D. Künzel J. (2014). Requirement for PBAF in Transcriptional Repression and Repair at DNA Breaks in Actively Transcribed Regions of Chromatin. Mol. Cel 55, 723–732. 10.1016/j.molcel.2014.06.028 PMC4157577 25066234

[B85] Kalb R. Mallery D. L. Larkin C. Huang J. T. J. Hiom K. (2014). BRCA1 Is a Histone-h2a-specific Ubiquitin Ligase. Cel Rep. 8, 999–1005. 10.1016/j.celrep.2014.07.025 PMC4382519 25131202

[B86] Kalocsay M. Hiller N. J. Jentsch S. (2009). Chromosome-wide Rad51 Spreading and SUMO-H2A.Z-dependent Chromosome Fixation in Response to a Persistent DNA Double-Strand Break. Mol. Cel 33, 335–343. 10.1016/j.molcel.2009.01.016 19217407

[B87] Kari V. Mansour W. Y. Raul S. K. Baumgart S. J. Mund A. Grade M. (2016). Loss of CHD1 Causes DNA Repair Defects and Enhances Prostate Cancer Therapeutic Responsiveness. EMBO Rep. 17, 1609–1623. 10.15252/embr.201642352 27596623 PMC5090703

[B88] Kent N. A. Chambers A. L. Downs J. A. (2007). Dual Chromatin Remodeling Roles for RSC during DNA Double Strand Break Induction and Repair at the Yeast MAT Locus. J. Biol. Chem. 282, 27693–27701. 10.1074/jbc.m704707200 17652077

[B89] Kilic S. Lezaja A. Gatti M. Bianco E. Michelena J. Imhof R. (2019). Phase Separation of 53 BP 1 Determines Liquid‐like Behavior of DNA Repair Compartments. Embo J. 38, e101379. 10.15252/embj.2018101379 31267591 PMC6694294

[B90] Krietenstein N. Wal M. Watanabe S. Park B. Peterson C. L. Pugh B. F. (2016). Genomic Nucleosome Organization Reconstituted with Pure Proteins. Cell 167, 709–721.e12. 10.1016/j.cell.2016.09.045 27768892 PMC5240917

[B91] Krogan N. J. Keogh M.-C. Datta N. Sawa C. Ryan O. W. Ding H. (2003). A Snf2 Family ATPase Complex Required for Recruitment of the Histone H2A Variant Htz1. Mol. Cel 12, 1565–1576. 10.1016/s1097-2765(03)00497-0 14690608

[B92] Kubik S. Bruzzone M. J. Challal D. Dreos R. Mattarocci S. Bucher P. (2019). Opposing Chromatin Remodelers Control Transcription Initiation Frequency and Start Site Selection. Nat. Struct. Mol. Biol. 26, 744–754. 10.1038/s41594-019-0273-3 31384063

[B93] Kubik S. O’Duibhir E. de Jonge W. J. Mattarocci S. Albert B. Falcone J.-L. (2018). Sequence-Directed Action of RSC Remodeler and General Regulatory Factors Modulates +1 Nucleosome Position to Facilitate Transcription. Mol. Cel 71, 89–102.e5. 10.1016/j.molcel.2018.05.030 29979971

[B94] Lademann C. A. Renkawitz J. Pfander B. Jentsch S. (2017). The INO80 Complex Removes H2A.Z to Promote Presynaptic Filament Formation during Homologous Recombination. Cel Rep. 19, 1294–1303. 10.1016/j.celrep.2017.04.051 28514650

[B95] Lan L. Ui A. Nakajima S. Hatakeyama K. Hoshi M. Watanabe R. (2010). The ACF1 Complex Is Required for DNA Double-Strand Break Repair in Human Cells. Mol. Cel 40, 976–987. 10.1016/j.molcel.2010.12.003 21172662

[B96] Larsen D. H. Poinsignon C. Gudjonsson T. Dinant C. Payne M. R. Hari F. J. (2010). The Chromatin-Remodeling Factor CHD4 Coordinates Signaling and Repair after DNA Damage. J. Cel Biol 190, 731–740. 10.1083/jcb.200912135 PMC2935572 20805324

[B97] Lazzaro F. Sapountzi V. Granata M. Pellicioli A. Vaze M. Haber J. E. (2008). Histone Methyltransferase Dot1 and Rad9 Inhibit Single-Stranded DNA Accumulation at DSBs and Uncapped Telomeres. Embo J. 27, 1502–1512. 10.1038/emboj.2008.81 18418382 PMC2328446

[B98] Lee H.-S. Park J.-H. Kim S.-J. Kwon S.-J. Kwon J. (2010). A Cooperative Activation Loop Among SWI/SNF, γ-H2AX and H3 Acetylation for DNA Double-Strand Break Repair. Embo J. 29, 1434–1445. 10.1038/emboj.2010.27 20224553 PMC2868568

[B99] Leung J. W. C. Makharashvili N. Agarwal P. Chiu L.-Y. Pourpre R. Cammarata M. B. (2017). ZMYM3 Regulates BRCA1 Localization at Damaged Chromatin to Promote DNA Repair. Genes Dev. 31, 260–274. 10.1101/gad.292516.116 28242625 PMC5358723

[B100] Lewis T. S. Sokolova V. Jung H. Ng H. Tan D. (2021). Structural Basis of Chromatin Regulation by Histone Variant H2A.Z. Nucleic Acids Res. 49, 11379–11391. 10.1093/nar/gkab907 34643712 PMC8565303

[B101] Li M. Hada A. Sen P. Olufemi L. Hall M. A. Smith B. Y. (2015). Dynamic Regulation of Transcription Factors by Nucleosome Remodeling. eLife Sci. 4, e06249. 10.7554/elife.06249 PMC4456607 26047462

[B102] Liang B. Qiu J. Ratnakumar K. Laurent B. C. (2007). RSC Functions as an Early Double-Strand-Break Sensor in the Cell's Response to DNA Damage. Curr. Biol. 17, 1432–1437. 10.1016/j.cub.2007.07.035 17689960 PMC2000454

[B103] Luger K. Dechassa M. L. Tremethick D. J. (2012). New Insights into Nucleosome and Chromatin Structure: an Ordered State or a Disordered Affair. Nat. Rev. Mol. Cel Biol 13, 436–447. 10.1038/nrm3382 PMC3408961 22722606

[B104] Luijsterburg M. S. Acs K. Ackermann L. Wiegant W. W. Bekker-Jensen S. Larsen D. H. (2012). A New Non-catalytic Role for Ubiquitin Ligase RNF8 in Unfolding Higher-Order Chromatin Structure. EMBO J. 31, 2511–2527. 10.1038/emboj.2012.104 22531782 PMC3365417

[B105] Luijsterburg M. S. de Krijger I. Wiegant W. W. Shah R. G. Smeenk G. de Groot A. J. L. (2016). PARP1 Links CHD2-Mediated Chromatin Expansion and H3.3 Deposition to DNA Repair by Non-homologous End-Joining. Mol. Cel 61, 547–562. 10.1016/j.molcel.2016.01.019 PMC4769320 26895424

[B106] Luk E. Ranjan A. FitzGerald P. C. Mizuguchi G. Huang Y. Wei D. (2010). Stepwise Histone Replacement by SWR1 Requires Dual Activation with Histone H2A.Z and Canonical Nucleosome. Cell 143, 725–736. 10.1016/j.cell.2010.10.019 21111233 PMC7251641

[B107] Ma N. F. Hu L. Fung J. M. Xie D. Zheng B. J. Chen L. (2008). Isolation and Characterization of a Novel Oncogene, Amplified in Liver Cancer 1, within a Commonly Amplified Region at 1q21 in Hepatocellular Carcinoma. Hepatology 47, 503–510. 10.1002/hep.22072 18023026

[B108] Marfella C. G. A. Imbalzano A. N. (2007). The Chd Family of Chromatin Remodelers. Mutat. Research/Fundamental Mol. Mech. Mutagenesis 618, 30–40. 10.1016/j.mrfmmm.2006.07.012 PMC1899158 17350655

[B109] Marini F. Rawal C. C. Liberi G. Pellicioli A. (2019). Regulation of DNA Double Strand Breaks Processing: Focus on Barriers. Front. Mol. Biosci. 6. 10.3389/fmolb.2019.00055 PMC6646425 31380392

[B208] Markert J. Zhou K. Luger K. (2021). SMARCAD1 is an ATP-Dependent Histone Octamer Exchange Factor with De Novo Nucleosome Assembly Activity. Sci. Adv. 7. 10.1126/sciadv.abk2380 PMC8519567 34652950

[B110] Meisenberg C. Pinder S. I. Hopkins S. R. Wooller S. K. Benstead-Hume G. Pearl F. M. G. (2019). Repression of Transcription at DNA Breaks Requires Cohesin throughout Interphase and Prevents Genome Instability. Mol. Cel 73, 212–223.e7. 10.1016/j.molcel.2018.11.001 PMC6344341 30554942

[B111] Mimitou E. P. Symington L. S. (2008). Sae2, Exo1 and Sgs1 Collaborate in DNA Double-Strand Break Processing. Nature 455, 770–774. 10.1038/nature07312 18806779 PMC3818707

[B112] Mimitou E. P. Yamada S. Keeney S. (2017). A Global View of Meiotic Double-Strand Break End Resection. Science 355, 40–45. 10.1126/science.aak9704 28059759 PMC5234563

[B113] Mittal P. Roberts C. W. M. (2020). The SWI/SNF Complex in Cancer - Biology, Biomarkers and Therapy. Nat. Rev. Clin. Oncol. 17, 435–448. 10.1038/s41571-020-0357-3 32303701 PMC8723792

[B114] Mizuguchi G. Shen X. Landry J. Wu W.-H. Sen S. Wu C. (2004). ATP-driven Exchange of Histone H2AZ Variant Catalyzed by SWR1 Chromatin Remodeling Complex. Science 303, 343–348. 10.1126/science.1090701 14645854

[B115] Morillo-Huesca M. Clemente-Ruiz M. Andújar E. Prado F. (2010). The SWR1 Histone Replacement Complex Causes Genetic Instability and Genome-wide Transcription Misregulation in the Absence of H2A.Z. PLoS One 5, e12143. 10.1371/journal.pone.0012143 20711347 PMC2920830

[B116] Morrison A. J. Highland J. Krogan N. J. Arbel-Eden A. Greenblatt J. F. Haber J. E. (2004). INO80 and γ-H2AX Interaction Links ATP-dependent Chromatin Remodeling to DNA Damage Repair. Cell 119, 767–775. 10.1016/j.cell.2004.11.037 15607974

[B117] Myler L. R. Gallardo I. F. Soniat M. M. Deshpande R. A. Gonzalez X. B. Kim Y. (2017). Single-Molecule Imaging Reveals How Mre11-Rad50-Nbs1 Initiates DNA Break Repair. Mol. Cel 67, 891–898.e4. 10.1016/j.molcel.2017.08.002 PMC5609712 28867292

[B118] Nagai S. Dubrana K. Tsai-Pflugfelder M. Davidson M. B. Roberts T. M. Brown G. W. (2008). Functional Targeting of DNA Damage to a Nuclear Pore-Associated SUMO-dependent Ubiquitin Ligase. Science 322, 597–602. 10.1126/science.1162790 18948542 PMC3518492

[B119] Nakamura K. Kato A. Kobayashi J. Yanagihara H. Sakamoto S. Oliveira D. V. N. P. (2011). Regulation of Homologous Recombination by RNF20-dependent H2B Ubiquitination. Mol. Cel 41, 515–528. 10.1016/j.molcel.2011.02.002 21362548

[B120] Nakamura K. Saredi G. Becker J. R. Foster B. M. Nguyen N. V. Beyer T. E. (2019). H4K20me0 Recognition by BRCA1-BARD1 Directs Homologous Recombination to Sister Chromatids. Nat. Cel Biol 21, 311–318. 10.1038/s41556-019-0282-9 PMC6420097 30804502

[B121] Nakamura T. M. Du L.-L. Redon C. Russell P. (2004). Histone H2A Phosphorylation Controls Crb2 Recruitment at DNA Breaks, Maintains Checkpoint Arrest, and Influences DNA Repair in Fission Yeast. Mol. Cel Biol 24, 6215–6230. 10.1128/MCB.24.14.6215-6230.2004 PMC434244 15226425

[B122] Nishibuchi I. Suzuki H. Kinomura A. Sun J. Liu N.-A. Horikoshi Y. (2014). Reorganization of Damaged Chromatin by the Exchange of Histone Variant H2A.Z-2. Int. J. Radiat. Oncology*Biology*Physics 89, 736–744. 10.1016/j.ijrobp.2014.03.031 24969791

[B123] Niu H. Chung W.-H. Zhu Z. Kwon Y. Zhao W. Chi P. (2010). Mechanism of the ATP-dependent DNA End-Resection Machinery from *Saccharomyces cerevisiae* . Nature 467, 108–111. 10.1038/nature09318 20811460 PMC2955862

[B124] Oberbeckmann E. Krietenstein N. Niebauer V. Wang Y. Schall K. Moldt M. (2021a). Genome Information Processing by the INO80 Chromatin Remodeler Positions Nucleosomes. Nat. Commun. 12, 3231. 10.1038/s41467-021-23016-z 34050142 PMC8163841

[B125] Oberbeckmann E. Niebauer V. Watanabe S. Farnung L. Moldt M. Schmid A. (2021b). Ruler Elements in Chromatin Remodelers Set Nucleosome Array Spacing and Phasing. Nat. Commun. 12, 3232. 10.1038/s41467-021-23015-0 34050140 PMC8163753

[B126] Ocampo J. Chereji R. V. Eriksson P. R. Clark D. J. (2016). The ISW1 and CHD1 ATP-dependent Chromatin Remodelers Compete to Set Nucleosome Spacingin Vivo. Nucleic Acids Res. 44, 4625–4635. 10.1093/nar/gkw068 26861626 PMC4889916

[B127] Ogiwara H. Ui A. Otsuka A. Satoh H. Yokomi I. Nakajima S. (2011). Histone Acetylation by CBP and P300 at Double-Strand Break Sites Facilitates SWI/SNF Chromatin Remodeling and the Recruitment of Non-homologous End Joining Factors. Oncogene 30, 2135–2146. 10.1038/onc.2010.592 21217779

[B128] Oza P. Jaspersen S. L. Miele A. Dekker J. Peterson C. L. (2009). Mechanisms that Regulate Localization of a DNA Double-Strand Break to the Nuclear Periphery. Genes Dev. 23, 912–927. 10.1101/gad.1782209 19390086 PMC2675867

[B129] Pan M.-R. Hsieh H.-J. Dai H. Hung W.-C. Li K. Peng G. (2012). Chromodomain Helicase DNA-Binding Protein 4 (CHD4) Regulates Homologous Recombination DNA Repair, and its Deficiency Sensitizes Cells to poly(ADP-Ribose) Polymerase (PARP) Inhibitor Treatment. J. Biol. Chem. 287, 6764–6772. 10.1074/jbc.M111.287037 22219182 PMC3307306

[B130] Panier S. Boulton S. J. (2014). Double-strand Break Repair: 53BP1 Comes into Focus. Nat. Rev. Mol. Cel Biol 15, 7–18. 10.1038/nrm3719 24326623

[B131] Papamichos-Chronakis M. Krebs J. E. Peterson C. L. (2006). Interplay between Ino80 and Swr1 Chromatin Remodeling Enzymes Regulates Cell Cycle Checkpoint Adaptationin Response to DNA Damage. Genes Dev. 20, 2437–2449. 10.1101/gad.1440206 16951256 PMC1560417

[B132] Papamichos-Chronakis M. Watanabe S. Rando O. J. Peterson C. L. (2011). Global Regulation of H2A.Z Localization by the INO80 Chromatin-Remodeling Enzyme Is Essential for Genome Integrity. Cell 144, 200–213. 10.1016/j.cell.2010.12.021 21241891 PMC3035940

[B133] Park J.-H. Park E.-J. Lee H.-S. Kim S. J. Hur S.-K. Imbalzano A. N. (2006). Mammalian SWI/SNF Complexes Facilitate DNA Double-Strand Break Repair by Promoting γ-H2AX Induction. Embo J. 25, 3986–3997. 10.1038/sj.emboj.7601291 16932743 PMC1560357

[B134] Peng G. Yim E.-K. Dai H. Jackson A. P. Burgt I. V. D. Pan M.-R. (2009). BRIT1/MCPH1 Links Chromatin Remodelling to DNA Damage Response. Nat. Cel Biol 11, 865–872. 10.1038/ncb1895 PMC2714531 19525936

[B135] Peritore M. Reusswig K.-U. Bantele S. C. S. Straub T. Pfander B. (2021). Strand-specific ChIP-Seq at DNA Breaks Distinguishes ssDNA versus dsDNA Binding and Refutes Single-Stranded Nucleosomes. Mol. Cel 81, 1841–1853.e4. 10.1016/j.molcel.2021.02.005 33651987

[B136] Pessina F. Lowndes N. F. (2014). The RSF1 Histone-Remodelling Factor Facilitates DNA Double-Strand Break Repair by Recruiting Centromeric and Fanconi Anaemia Proteins. Plos Biol. 12, e1001856. 10.1371/journal.pbio.1001856 24800743 PMC4011676

[B137] Pfander B. Diffley J. F. X. (2011). Dpb11 Coordinates Mec1 Kinase Activation with Cell Cycle-Regulated Rad9 Recruitment. EMBO J. 30, 4897–4907. 10.1038/emboj.2011.345 21946560 PMC3243626

[B138] Polo S. E. Kaidi A. Baskcomb L. Galanty Y. Jackson S. P. (2010). Regulation of DNA-Damage Responses and Cell-Cycle Progression by the Chromatin Remodelling Factor CHD4. Embo J. 29, 3130–3139. 10.1038/emboj.2010.188 20693977 PMC2944064

[B139] Qi W. Chen H. Xiao T. Wang R. Li T. Han L. (2016). Acetyltransferase P300 Collaborates with Chromodomain Helicase DNA-Binding Protein 4 (CHD4) to Facilitate DNA Double-Strand Break Repair. Mutage 31, 193–203. 10.1093/mutage/gev075 26546801

[B140] Qi W. Wang R. Chen H. Wang X. Xiao T. Boldogh I. (2015). BRG1 Promotes DNA Double-Strand Break Repair by Facilitating the Replacement of RPA with RAD51. J. Cel Sci. 128, 317–330. 10.1242/jcs.159103 PMC4294775 25395584

[B141] Qiu H. Biernat E. Govind C. K. Rawal Y. Chereji R. V. Clark D. J. (2020). Chromatin Remodeler Ino80C Acts Independently of H2A.Z to Evict Promoter Nucleosomes and Stimulate Transcription of Highly Expressed Genes in Yeast. Nucleic Acids Res. 48, 8408–8430. 10.1093/nar/gkaa571 32663283 PMC7470979

[B142] Ranjan A. Wang F. Mizuguchi G. Wei D. Huang Y. Wu C. (2015). H2A Histone-fold and DNA Elements in Nucleosome Activate SWR1-Mediated H2A.Z Replacement in Budding Yeast. eLife Sci. 4, e06845. 10.7554/eLife.06845 PMC4508883 26116819

[B143] Ranjha L. Howard S. M. Cejka P. (2018). Main Steps in DNA Double-Strand Break Repair: an Introduction to Homologous Recombination and Related Processes. Chromosoma 127, 187–214. 10.1007/s00412-017-0658-1 29327130

[B144] Reginato G. Cannavo E. Cejka P. (2017). Physiological Protein Blocks Direct the Mre11-Rad50-Xrs2 and Sae2 Nuclease Complex to Initiate DNA End Resection. Genes Dev. 31, 2325–2330. 10.1101/gad.308254.117 29321179 PMC5795779

[B145] Rossi M. J. Lai W. K. M. Pugh B. F. (2018). Genome-wide Determinants of Sequence-specific DNA Binding of General Regulatory Factors. Genome Res. 28, 497–508. 10.1101/gr.229518.117 29563167 PMC5880240

[B146] Rother M. B. Pellegrino S. Smith R. Gatti M. Meisenberg C. Wiegant W. W. (2020). CHD7 and 53BP1 Regulate Distinct Pathways for the Re-ligation of DNA Double-Strand Breaks. Nat. Commun. 11, 1–19. 10.1038/s41467-020-19502-5 33188175 PMC7666215

[B147] Ryu T. Spatola B. Delabaere L. Bowlin K. Hopp H. Kunitake R. (2015). Heterochromatic Breaks Move to the Nuclear Periphery to Continue Recombinational Repair. Nat. Cel Biol 17, 1401–1411. 10.1038/ncb3258 PMC4628585 26502056

[B148] Sanchez A. Lee D. Kim D. I. Miller K. M. (2021). Making Connections: Integrative Signaling Mechanisms Coordinate DNA Break Repair in Chromatin. Front. Genet. 12. 10.3389/fgene.2021.747734 PMC8514019 34659365

[B149] Sánchez-Molina S. Mortusewicz O. Bieber B. Auer S. Eckey M. Leonhardt H. (2011). Role for hACF1 in the G2/M Damage Checkpoint. Nucleic Acids Res. 39, 8445–8456. 10.1093/nar/gkr435 21745822 PMC3201854

[B150] Sanders S. L. Portoso M. Mata J. Bähler J. Allshire R. C. Kouzarides T. (2004). Methylation of Histone H4 Lysine 20 Controls Recruitment of Crb2 to Sites of DNA Damage. Cell 119, 603–614. 10.1016/j.cell.2004.11.009 15550243

[B151] Santisteban M. S. Kalashnikova T. Smith M. M. (2000). Histone H2A.Z Regulates Transcription and Is Partially Redundant with Nucleosome Remodeling Complexes. Cell 103, 411–422. 10.1016/S0092-8674(00)00133-1 11081628

[B152] Sartori A. A. Lukas C. Coates J. Mistrik M. Fu S. Bartek J. (2007). Human CtIP Promotes DNA End Resection. Nature 450, 509–514. 10.1038/nature06337 17965729 PMC2409435

[B153] Schrank B. R. Aparicio T. Li Y. Chang W. Chait B. T. Gundersen G. G. (2018). Nuclear ARP2/3 Drives DNA Break Clustering for Homology-Directed Repair. Nature 559, 61–66. 10.1038/s41586-018-0237-5 29925947 PMC6145447

[B154] Sellou H. Lebeaupin T. Chapuis C. Smith R. Hegele A. Singh H. R. (2016). The poly(ADP-ribose)-dependent Chromatin Remodeler Alc1 Induces Local Chromatin Relaxation upon DNA Damage. MBoC 27, 3791–3799. 10.1091/mbc.E16-05-0269 27733626 PMC5170603

[B155] Sheu J. J.-C. Guan B. Choi J.-H. Lin A. Lee C.-H. Hsiao Y.-T. (2010). Rsf-1, a Chromatin Remodeling Protein, Induces DNA Damage and Promotes Genomic Instability. J. Biol. Chem. 285, 38260–38269. 10.1074/jbc.M110.138735 20923775 PMC2992260

[B156] Shibata A. Moiani D. Arvai A. S. Perry J. Harding S. M. Genois M.-M. (2014). DNA Double-Strand Break Repair Pathway Choice Is Directed by Distinct MRE11 Nuclease Activities. Mol. Cel 53, 7–18. 10.1016/j.molcel.2013.11.003 PMC3909494 24316220

[B157] Shim E. Y. Hong S. J. Oum J.-H. Yanez Y. Zhang Y. Lee S. E. (2007). RSC Mobilizes Nucleosomes to Improve Accessibility of Repair Machinery to the Damaged Chromatin. Mol. Cel Biol 27, 1602–1613. 10.1128/mcb.01956-06 ▿ † PMC1820475 17178837

[B158] Shim E. Y. Ma J.-L. Oum J.-H. Yanez Y. Lee S. E. (2005). The Yeast Chromatin Remodeler RSC Complex Facilitates End Joining Repair of DNA Double-Strand Breaks. Mol. Cel Biol 25, 3934–3944. 10.1128/mcb.25.10.3934-3944.2005 PMC1087737 15870268

[B159] Singh R. K. Fan J. Gioacchini N. Watanabe S. Bilsel O. Peterson C. L. (2019). Transient Kinetic Analysis of SWR1C-Catalyzed H2A.Z Deposition Unravels the Impact of Nucleosome Dynamics and the Asymmetry of Histone Exchange. Cel Rep. 27, 374–386. e4. 10.1016/j.celrep.2019.03.035 PMC6545893 30970243

[B160] Smeenk G. van Attikum H. (2013). The Chromatin Response to DNA Breaks: Leaving a Mark on Genome Integrity. Annu. Rev. Biochem. 82, 55–80. 10.1146/annurev-biochem-061809-174504 23414304

[B161] Smeenk G. Wiegant W. W. Marteijn J. A. Luijsterburg M. S. Sroczynski N. Costelloe T. (2012). Poly(ADP-ribosyl)ation Links the Chromatin Remodeler SMARCA5/SNF2H to RNF168-dependent DNA Damage Signaling. J. Cel Sci. 126, 889–903. 10.1242/jcs.109413 23264744

[B162] Smeenk G. Wiegant W. W. Vrolijk H. Solari A. P. Pastink A. van Attikum H. (2010). The NuRD Chromatin-Remodeling Complex Regulates Signaling and Repair of DNA Damage. J. Cel Biol 190, 741–749. 10.1083/jcb.201001048 PMC2935570 20805320

[B163] Smith R. Sellou H. Chapuis C. Huet S. Timinszky G. (2018). CHD3 and CHD4 Recruitment and Chromatin Remodeling Activity at DNA Breaks Is Promoted by Early poly(ADP-ribose)-dependent Chromatin Relaxation. Nucleic Acids Res. 46, 6087–6098. 10.1093/nar/gky334 29733391 PMC6158744

[B164] Spruijt C. G. Luijsterburg M. S. Menafra R. Lindeboom R. G. H. Jansen P. W. T. C. Edupuganti R. R. (2016). ZMYND8 Co-localizes with NuRD on Target Genes and Regulates Poly(ADP-ribose)-dependent Recruitment of GATAD2A/NuRD to Sites of DNA Damage. Cel Rep. 17, 783–798. 10.1016/j.celrep.2016.09.037 27732854

[B165] Symington L. S. Gautier J. (2011). Double-strand Break End Resection and Repair Pathway Choice. Annu. Rev. Genet. 45, 247–271. 10.1146/annurev-genet-110410-132435 21910633

[B166] Symington L. S. (2016). Mechanism and Regulation of DNA End Resection in Eukaryotes. Crit. Rev. Biochem. Mol. Biol. 51, 195–212. 10.3109/10409238.2016.1172552 27098756 PMC4957645

[B167] Talbert P. B. Henikoff S. (2010). Histone Variants - Ancient Wrap Artists of the Epigenome. Nat. Rev. Mol. Cel Biol 11, 264–275. 10.1038/nrm2861 20197778

[B168] Talbert P. B. Henikoff S. (2021). Histone Variants at a Glance. J. Cel Sci. 134, jcs244749. 10.1242/jcs.244749 PMC8015243 33771851

[B169] Talbert P. B. Henikoff S. (2017). Histone Variants on the Move: Substrates for Chromatin Dynamics. Nat. Rev. Mol. Cel Biol 18, 115–126. 10.1038/nrm.2016.148 27924075

[B170] Toh G. W.-L. O'Shaughnessy A. M. Jimeno S. Dobbie I. M. Grenon M. Maffini S. (2006). Histone H2A Phosphorylation and H3 Methylation Are Required for a Novel Rad9 DSB Repair Function Following Checkpoint Activation. DNA Repair 5, 693–703. 10.1016/j.dnarep.2006.03.005 16650810

[B171] Toiber D. Erdel F. Bouazoune K. Silberman D. M. Zhong L. Mulligan P. (2013). SIRT6 Recruits SNF2H to DNA Break Sites, Preventing Genomic Instability through Chromatin Remodeling. Mol. Cel 51, 454–468. 10.1016/j.molcel.2013.06.018 PMC3761390 23911928

[B172] Tong J. K. Hassig C. A. Schnitzler G. R. Kingston R. E. Schreiber S. L. (1998). Chromatin Deacetylation by an ATP-dependent Nucleosome Remodelling Complex. Nature 395, 917–921. 10.1038/27699 9804427

[B173] Tramantano M. Sun L. Au C. Labuz D. Liu Z. Chou M. (2016). Constitutive Turnover of Histone H2A.Z at Yeast Promoters Requires the Preinitiation Complex. eLife Sci. 5, e14243. 10.7554/eLife.14243 PMC4995100 27438412

[B174] Tripuraneni V. Memisoglu G. MacAlpine H. K. Tran T. Q. Zhu W. Hartemink A. J. (2021). Local Nucleosome Dynamics and Eviction Following a Double-Strand Break Are Reversible by NHEJ-Mediated Repair in the Absence of DNA Replication. Genome Res. 31, 775–788. 10.1101/gr.271155.120 33811083 PMC8092003

[B175] Tsabar M. Hicks W. M. Tsaponina O. Haber J. E. (2016). Re-establishment of Nucleosome Occupancy during Double-Strand Break Repair in Budding Yeast. DNA Repair 47, 21–29. 10.1016/j.dnarep.2016.09.005 27720308 PMC5159207

[B176] Tsouroula K. Furst A. Rogier M. Heyer V. Maglott-Roth A. Ferrand A. (2016). Temporal and Spatial Uncoupling of DNA Double Strand Break Repair Pathways within Mammalian Heterochromatin. Mol. Cel 63, 293–305. 10.1016/j.molcel.2016.06.002 27397684

[B177] Tsukuda T. Fleming A. B. Nickoloff J. A. Osley M. A. (2005). Chromatin Remodelling at a DNA Double-Strand Break Site in *Saccharomyces cerevisiae* . Nature 438, 379–383. 10.1038/nature04148 16292314 PMC1388271

[B178] Tsukuda T. Lo Y.-C. Krishna S. Sterk R. Osley M. A. Nickoloff J. A. (2009). INO80-dependent Chromatin Remodeling Regulates Early and Late Stages of Mitotic Homologous Recombination. DNA Repair 8, 360–369. 10.1016/j.dnarep.2008.11.014 19095087

[B179] Ui A. Ogiwara H. Nakajima S. Kanno S. Watanabe R. Harata M. (2014). Possible Involvement of LKB1-AMPK Signaling in Non-homologous End Joining. Oncogene 33, 1640–1648. 10.1038/onc.2013.125 23584481 PMC6508539

[B180] van Attikum H. Fritsch O. Gasser S. M. (2007). Distinct Roles for SWR1 and INO80 Chromatin Remodeling Complexes at Chromosomal Double-Strand Breaks. Embo J. 26, 4113–4125. 10.1038/sj.emboj.7601835 17762868 PMC2230671

[B181] van Attikum H. Fritsch O. Hohn B. Gasser S. M. (2004). Recruitment of the INO80 Complex by H2A Phosphorylation Links ATP-dependent Chromatin Remodeling with DNA Double-Strand Break Repair. Cell 119, 777–788. 10.1016/j.cell.2004.11.033 15607975

[B182] Van H. T. Santos M. A. (2018). Histone Modifications and the DNA Double-Strand Break Response. Cell Cycle 17, 2399–2410. 10.1080/15384101.2018.1542899 30394812 PMC6342081

[B183] Venkatesh S. Workman J. L. (2015). Histone Exchange, Chromatin Structure and the Regulation of Transcription. Nat. Rev. Mol. Cel Biol 16, 178–189. 10.1038/nrm3941 25650798

[B184] Vidi P.-A. Liu J. Salles D. Jayaraman S. Dorfman G. Gray M. (2014). NuMA Promotes Homologous Recombination Repair by Regulating the Accumulation of the ISWI ATPase SNF2h at DNA Breaks. Nucleic Acids Res. 42, 6365–6379. 10.1093/nar/gku296 24753406 PMC4041463

[B185] Wang F. Ranjan A. Wei D. Wu C. (2016). Comment on "A Histone Acetylation Switch Regulates H2A.Z Deposition by the SWR-C Remodeling Enzyme". Science 353, 358. 10.1126/science.aad5921 27463665

[B186] Wang W. Daley J. M. Kwon Y. Krasner D. S. Sung P. (2017). Plasticity of the Mre11-Rad50-Xrs2-Sae2 Nuclease Ensemble in the Processing of DNA-Bound Obstacles. Genes Dev. 31, 2331–2336. 10.1101/gad.307900.117 29321177 PMC5795780

[B187] Watanabe R. Ui A. Kanno S.-I. Ogiwara H. Nagase T. Kohno T. (2014). SWI/SNF Factors Required for Cellular Resistance to DNA Damage Include ARID1A and ARID1B and Show Interdependent Protein Stability. Cancer Res. 74, 2465–2475. 10.1158/0008-5472.CAN-13-3608 24788099

[B188] Watanabe S. Peterson C. L. (2016). Response to Comment on "A Histone Acetylation Switch Regulates H2A.Z Deposition by the SWR-C Remodeling Enzyme". Science 353, 358. 10.1126/science.aad6398 PMC8121161 27463666

[B189] Watanabe S. Radman-Livaja M. Rando O. J. Peterson C. L. (2013). A Histone Acetylation Switch Regulates H2A.Z Deposition by the SWR-C Remodeling Enzyme. Science 340, 195–199. 10.1126/science.1229758 23580526 PMC3727404

[B190] Weiner A. Hughes A. Yassour M. Rando O. J. Friedman N. (2010). High-resolution Nucleosome Mapping Reveals Transcription-dependent Promoter Packaging. Genome Res. 20, 90–100. 10.1101/gr.098509.109 19846608 PMC2798834

[B191] Wiechens N. Singh V. Gkikopoulos T. Schofield P. Rocha S. Owen-Hughes T. (2016). The Chromatin Remodelling Enzymes SNF2H and SNF2L Position Nucleosomes Adjacent to CTCF and Other Transcription Factors. Plos Genet. 12, e1005940. 10.1371/journal.pgen.1005940 27019336 PMC4809547

[B192] Wiest N. E. Houghtaling S. Sanchez J. C. Tomkinson A. E. Osley M. A. (2017). The SWI/SNF ATP-dependent Nucleosome Remodeler Promotes Resection Initiation at a DNA Double-Strand Break in Yeast. Nucleic Acids Res. 45, 5887–5900. 10.1093/nar/gkx221 28398510 PMC5449591

[B193] WillhoftO.GhoneimM.LinC.-L.ChuaE. Y. D.WilkinsonM.ChabanY. (2018). Structure and Dynamics of the Yeast SWR1-Nucleosome Complex. Science 362, eaat7716. 10.1126/science.aat7716 30309918

[B194] Willhoft O. Wigley D. B. (2020). INO80 and SWR1 Complexes: the Non-identical Twins of Chromatin Remodelling. Curr. Opin. Struct. Biol. 61, 50–58. 10.1016/j.sbi.2019.09.002 31838293 PMC7171469

[B195] Wilson M. D. Benlekbir S. Fradet-Turcotte A. Sherker A. Julien J.-P. McEwan A. (2016). The Structural Basis of Modified Nucleosome Recognition by 53BP1. Nature 536, 100–103. 10.1038/nature18951 27462807

[B196] Wu W.-H. Wu C.-H. Ladurner A. Mizuguchi G. Wei D. Xiao H. (2009). N Terminus of Swr1 Binds to Histone H2AZ and Provides a Platform for Subunit Assembly in the Chromatin Remodeling Complex. J. Biol. Chem. 284, 6200–6207. 10.1074/jbc.M808830200 19088068 PMC2649089

[B197] Wysocki R. Javaheri A. Allard S. Sha F. Côté J. Kron S. J. (2005). Role of Dot1-dependent Histone H3 Methylation in G 1 and S Phase DNA Damage Checkpoint Functions of Rad9. Mol. Cel Biol 25, 8430–8443. 10.1128/MCB.25.19.8430-8443.2005 PMC1265753 16166626

[B198] Xiao A. Li H. Shechter D. Ahn S. H. Fabrizio L. A. Erdjument-Bromage H. (2009). WSTF Regulates the H2A.X DNA Damage Response via a Novel Tyrosine Kinase Activity. Nature 457, 57–62. 10.1038/nature07668 19092802 PMC2854499

[B199] Xu Y. Ayrapetov M. K. Xu C. Gursoy-Yuzugullu O. Hu Y. Price B. D. (2012). Histone H2A.Z Controls a Critical Chromatin Remodeling Step Required for DNA Double-Strand Break Repair. Mol. Cel 48, 723–733. 10.1016/j.molcel.2012.09.026 PMC3525728 23122415

[B200] Xue Y. Wong J. Moreno G. T. Young M. K. Côté J. Wang W. (1998). NURD, a Novel Complex with Both ATP-dependent Chromatin-Remodeling and Histone Deacetylase Activities. Mol. Cel 2, 851–861. 10.1016/s1097-2765(00)80299-3 9885572

[B201] Yamada K. Frouws T. D. Angst B. Fitzgerald D. J. DeLuca C. Schimmele K. (2011). Structure and Mechanism of the Chromatin Remodelling Factor ISW1a. Nature 472, 448–453. 10.1038/nature09947 21525927

[B202] Yu Y. Deng Y. Reed S. H. Millar C. B. Waters R. (2013). Histone Variant Htz1 Promotes Histone H3 Acetylation to Enhance Nucleotide Excision Repair in Htz1 Nucleosomes. Nucleic Acids Res. 41, 9006–9019. 10.1093/nar/gkt688 23925126 PMC3799447

[B203] Yuan G.-C. Liu Y.-J. Dion M. F. Slack M. D. Wu L. F. Altschuler S. J. (2005). Genome-Scale Identification of Nucleosome Positions in *S. cerevisiae* . Science 309, 626–630. 10.1126/science.1112178 15961632

[B204] Zhang H. Roberts D. N. Cairns B. R. (2005). Genome-Wide Dynamics of Htz1, a Histone H2A Variant that Poises Repressed/Basal Promoters for Activation through Histone Loss. Cell 123, 219–231. 10.1016/j.cell.2005.08.036 16239141 PMC2788555

[B205] Zhang Y. LeRoy G. Seelig H.-P. Lane W. S. Reinberg D. (1998). The Dermatomyositis-specific Autoantigen Mi2 Is a Component of a Complex Containing Histone Deacetylase and Nucleosome Remodeling Activities. Cell 95, 279–289. 10.1016/s0092-8674(00)81758-4 9790534

[B206] Zhou J. Li J. Serafim R. B. Ketchum S. Ferreira C. G. Liu J. C. (2018). Human CHD1 Is Required for Early DNA-Damage Signaling and Is Uniquely Regulated by its N Terminus. Nucleic Acids Res. 46, 3891–3905. 10.1093/nar/gky128 29529298 PMC5934646

[B207] Zhu Z. Chung W.-H. Shim E. Y. Lee S. E. Ira G. (2008). Sgs1 Helicase and Two Nucleases Dna2 and Exo1 Resect DNA Double-Strand Break Ends. Cell 134, 981–994. 10.1016/j.cell.2008.08.037 18805091 PMC2662516

